# PLGA nanoparticles in otoprotection and inner ear regeneration: a new frontier in nanomedicine for hearing disorders

**DOI:** 10.1039/d5ra06007a

**Published:** 2025-12-22

**Authors:** Olusegun Oluwaseun Jimoh, Tolulope Ajuwon, Somtochukwu Samuel Okonkwo, Raymond Femi Awoyemi, Ibukunoluwa Olaosebikan, Olatayo Adedayo Olahanmi, Christopher Mbonu, Idris Oladimeji Junaid, Ikenna Odezuligbo, Kristinoba Olotu, Ikhazuagbe Hilary Ifijen

**Affiliations:** a Department of Pharmacology and Neuroscience, Southern Illinois University School of Medicine 801 N Rutledge Springfield Illinois 62702 USA godwinoluwaseun001@gmail.com; b South Dakota School of Mines and Technology USA; c Chemical and Bimolecular Engineering, University of Houston 4800 Calhoun Road Houston TX 77004 USA; d Department of Chemistry, Mississippi State University MS 39762 USA; e C. Eugene Bennett Department of Chemistry, West Virginia University 217 Clark Halll Morgantown WV 26506 USA; f Department of Chemistry, Middle Tennessee State University USA; g Department of Chemical Engineering & Material Science 1 Castle Point Terrace Hoboken New Jersey 07030 USA; h Quality Assurance Department, Senior QA Specialist Catalent, 7555 Harmans Rd, Harmans MD 21077 USA idris.junaid@catalent.com; i Department of Physics, Creighton University USA; j Department of Mechanical Engineering, University of Texas at Tyler USA; k Department of Research Outreach, Rubber Research Institute of Nigeria Iyanomo Benin Nigeria ifijen.hilary@rrin.gov.ng larylans4u@yahoo.com

## Abstract

Sensorineural hearing loss (SNHL) remains a significant global health burden due to its irreversible nature and limited treatment options. Recent advances in nanotechnology have introduced new possibilities for targeted drug delivery to the inner ear, with poly(lactic-*co*-glycolic acid) (PLGA) nanoparticles emerging as a promising platform. Biodegradable and biocompatible, PLGA nanoparticles can encapsulate diverse therapeutic agents and enable sustained, site-specific delivery to cochlear and vestibular structures. This review highlights key physicochemical properties of PLGA nanoparticles relevant to inner ear therapy, including controlled release, tunable surface modifications, and ideal particle size. It explores advanced cochlear targeting techniques—such as round window membrane penetration, magnetic navigation, and ultrasound-assisted delivery—and their applications in otoprotection against ototoxic drugs and noise-induced hearing loss. Additionally, the potential of PLGA nanoparticles in inner ear regeneration, particularly through hair cell restoration and stem cell-based repair, is examined. Preclinical evidence, comparative advantages over other nanocarriers, and challenges in clinical translation are critically reviewed. Finally, emerging trends are discussed, including integration with gene editing, combination therapies, and personalized medicine. This review underscores the transformative potential of PLGA-based nanomedicine in protecting and restoring hearing function, paving the way for future clinical breakthroughs.

## Abstract 2

Sensorineural hearing loss (SNHL) remains a significant global health burden due to its irreversible nature and limited treatment options.

## Graphical abstract

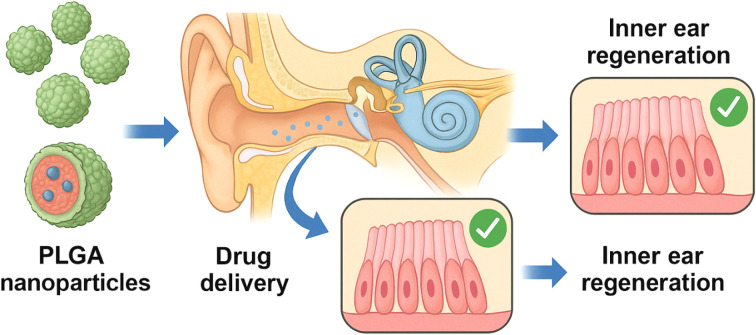

## 1. Introduction

Hearing loss is a pervasive and progressively debilitating condition that affects communication, social interaction, cognitive function, and overall quality of life. According to the World Health Organization, over 430 million people globally suffer from disabling hearing loss, with this number projected to exceed 700 million by 2050.^1,2^ Sensorineural hearing loss (SNHL), accounting for the majority of these cases, arises from irreversible damage to the sensory hair cells, synapses, or spiral ganglion neurons (SGNs) within the cochlea. This form of hearing impairment can result from aging (presbycusis), excessive noise exposure, ototoxic medications (*e.g.*, aminoglycosides, cisplatin), infections, or genetic factors.^3–5^

One of the major challenges in treating SNHL is the anatomical and physiological inaccessibility of the inner ear. The cochlea is encased within the dense temporal bone and protected by the blood-labyrinth barrier (BLB), which limits the penetration of systemically administered drugs. Furthermore, the cochlear microenvironment lacks intrinsic regenerative capacity, making damage largely permanent. These barriers necessitate the development of alternative, localized, and highly efficient delivery systems capable of transporting therapeutic agents directly to inner ear targets with minimal systemic exposure.^6–8^

Over the years, several strategies have been explored for otoprotection and inner ear regeneration beyond nanomedicine. These include the use of systemic and local pharmacological agents such as corticosteroids, antioxidants, and neurotrophic factors to mitigate inflammation, oxidative stress, and neural degeneration. Gene therapy approaches have also been employed to reintroduce or correct genes crucial for hair cell survival and synaptic repair, while RNA-based therapeutics and CRISPR/Cas9-mediated editing show promise in addressing hereditary hearing loss. Stem cell transplantation—using embryonic, induced pluripotent, or mesenchymal stem cells—has been investigated for its potential to repopulate lost sensory hair cells and spiral ganglion neurons, though challenges related to integration and functionality persist.^13–15^ Additionally, biomaterial-based scaffolds and hydrogel systems have been developed to provide structural support and controlled delivery of regenerative cues within the cochlear microenvironment. These methods represent important complementary avenues that inform and enhance the efficacy of nanoparticle-based interventions.

Nanomedicine has emerged as a revolutionary platform in biomedical research, offering innovative solutions for targeted drug delivery, sustained release, and enhanced cellular uptake.^9–13^ Among the various types of nanocarriers investigated, poly(lactic-*co*-glycolic acid) (PLGA)-based nanoparticles have gained substantial attention in the context of inner ear therapy. PLGA is an FDA- and EMA-approved biodegradable polymer with a well-established safety profile, capable of encapsulating a wide range of therapeutic molecules including small drugs, peptides, nucleic acids, and proteins.^14,15^

PLGA nanoparticles exhibit several key properties that make them highly suitable for inner ear applications. These include tunable degradation rates for controlled drug release, modifiable surface characteristics for enhanced permeability and target specificity, and sizes amenable to crossing the round window membrane (RWM)—the primary gateway for non-invasive cochlear access. By leveraging these attributes, PLGA nanoparticles have been employed to deliver antioxidants, anti-inflammatory agents, neurotrophic factors, and gene therapy components to mitigate ototoxicity, prevent neurodegeneration, and promote cochlear regeneration.^16–18^

This review provides a comprehensive overview of recent advances in the application of PLGA nanoparticles for otoprotection and inner ear regeneration. We begin by discussing the fundamental properties of PLGA nanoparticles relevant to cochlear drug delivery, followed by an exploration of administration routes and strategies for overcoming the anatomical barriers of the inner ear. The therapeutic potential of PLGA nanoparticles is then examined in the context of ototoxic drug exposure, noise-induced hearing loss, and autoimmune inner ear diseases. Finally, we highlight their emerging role in supporting hair cell regeneration, spiral ganglion neuron preservation, and stem cell integration, as well as the current limitations and future prospects of translating PLGA-based nanomedicine into clinical otology.

## 2. Properties of PLGA nanoparticles relevant to inner ear therapy

### 2.1 Biodegradability and biocompatibility

Poly(lactic-*co*-glycolic acid) (PLGA) is a well-established FDA and EMA-approved biodegradable copolymer composed of lactic and glycolic acid monomers, with extensive use in sutures, implants, and controlled-release drug delivery formulations.^19,20^ Upon administration, PLGA undergoes predictable bulk hydrolysis, cleaving ester linkages and yielding lactic and glycolic acids—metabolites readily incorporated into the Krebs cycle or excreted *via* renal pathways—thus minimizing systemic toxicity.^19,21^

The degradability of PLGA is highly tunable; by adjusting factors such as the lactic-to-glycolic acid ratio, polymer molecular weight, and end-group chemistry, release profiles can be tailored across days to months. In particular, the 50 : 50 lactic:glycolic acid ratio typically yields the fastest degradation (approx. 4–8 weeks), while higher lactic acid content enhances hydrophobicity and prolongs the degradation timeframe—making it ideal for sustained drug delivery in confined anatomical regions like the cochlea.^19,20^

One innovative application of PLGA in oto-therapeutics involves PLGA–PEG–PLGA thermosensitive hydrogels designed for intratympanic injection. In guinea pig models, Feng *et al.* demonstrated that 0.05 mL of such a hydrogel can sustain drug release over several days without affecting auditory brainstem response (ABR), whereas higher volumes (0.1 mL) induced hearing loss, underscoring the importance of dose optimization.^19^

Enhancement of inner ear delivery is further achieved by surface-modifying PLGA nanoparticles with hydrophilic excipients such as poloxamer 407 or PEG, which increase mucoadhesion and retention at the round window membrane (RWM).

These findings support the premise that PLGA-based systems offer a versatile and secure platform for inner ear drug delivery. Their tunable degradation kinetics, established safety profile, and compatibility with surface functionalization strategies make them ideally suited for long-term, localized therapeutic administration in the cochlea—offering a promising approach for treating sensorineural hearing conditions.

### 2.2 Controlled and sustained drug release

The ability of poly(lactic-*co*-glycolic acid) (PLGA) to modulate drug release kinetics hinges significantly on its compositional tunability and the physicochemical dynamics of its degradation, which are governed by factors such as the lactic-to-glycolic acid ratio, molecular weight, crystallinity, and the presence of end-capping groups.^22^ The lactic-to-glycolic acid ratio plays a central role in controlling the hydrophilicity and degradation rate of the polymer; for instance, a higher glycolic acid content increases the polymer's hydrophilicity, leading to faster water absorption and accelerated hydrolytic degradation. Conversely, a higher lactic acid content enhances hydrophobicity and slows down degradation, enabling a more sustained release of encapsulated drugs.

Molecular weight also critically influences the degradation profile—high-molecular-weight PLGA typically forms denser matrices with slower degradation rates and prolonged drug release, whereas low-molecular-weight PLGA degrades more rapidly due to increased surface area and chain-end exposure. Crystallinity further modulates water diffusion; highly crystalline regions act as barriers to water penetration, delaying hydrolysis and drug release, while amorphous regions facilitate faster degradation.

In addition, the presence of end-capping groups (*e.g.*, ester-terminated *vs.* acid-terminated chains) affects the polymer's susceptibility to hydrolysis. Acid-terminated PLGA absorbs water more readily and undergoes faster chain scission compared to ester-capped variants. These interdependent variables collectively influence the polymer matrix's interaction with its aqueous environment, regulating how quickly it absorbs water, undergoes hydrolytic chain cleavage, and erodes under physiological conditions—ultimately determining the duration, rate, and profile of therapeutic agent release.^23,24^

For example, Makadia and Siegel (2011) highlighted that altering the lactic-to-glycolic acid ratio can precisely influence the hydrophilicity, mechanical integrity, and degradation profile of PLGA-based drug delivery systems.^22^ This compositional flexibility enables researchers to design matrices with desired erosion profiles, ranging from rapid (weeks) to prolonged (months), depending on therapeutic needs. Specifically, PLGA with a 50 : 50 molar ratio of lactic to glycolic acid degrades most rapidly—often within four to eight weeks—due to its balanced hydrophilic properties, which favor water uptake and hydrolytic chain scission. Conversely, increasing the lactic acid content confers greater hydrophobicity and crystallinity, leading to slower degradation and sustained drug release profiles.

Expanding upon this, Danhier *et al.* (2012) corroborated that PLGA's versatility is further enhanced by its mechanical strength, biocompatibility, and approval by regulatory agencies such as the FDA.^23^ These properties have positioned PLGA as a frontrunner in drug delivery research, especially in systems designed for controlled release of small molecules, peptides, and proteins. Importantly, PLGA degrades *via* non-enzymatic hydrolysis of its ester bonds, resulting in bulk erosion where water permeates the matrix faster than the rate of degradation. This results in a complex release mechanism that combines surface and bulk erosion, surface and bulk diffusion, and autocatalytic hydrolysis due to accumulating carboxylic acid end-groups.

Ford *et al.* (2011), in a multi-scale modeling study, provided further mechanistic insights into the degradation and release behaviour of PLGA microparticles.^24^ They emphasized the role of matrix porosity, drug-polymer affinity, and particle size in dictating release kinetics.^24^ Their work demonstrated how drug release from PLGA devices often follows a biphasic or triphasic pattern.

The degradation profile of PLGA is influenced by multiple interrelated variables, including the polymer's molecular weight, crystallinity, glass transition temperature (*T*_g_), and drug loading. While the hydrolysis-driven erosion is the dominant mechanism, discrepancies between *in vitro* and *in vivo* degradation rates have led some researchers to hypothesize possible enzymatic contributions, though consensus remains elusive. Once degraded, PLGA's byproducts—lactic and glycolic acids—enter natural metabolic pathways, such as the tricarboxylic acid cycle, and are excreted as carbon dioxide and water, confirming the polymer's biocompatibility and safety. The integration of structural engineering and molecular design in PLGA-based systems facilitates the fabrication of highly tunable, clinically translatable drug delivery platforms. These systems not only offer precise control over release profiles but also maintain structural integrity under physiological stress—a critical factor in ensuring therapeutic efficacy. The growing body of mechanistic and computational studies, including those by Ford *et al.* (2011)^24^ and Danhier *et al.* (2012),^23^ continues to advance the understanding of PLGA dynamics, paving the way for innovative applications in fields such as inner ear therapy, oncology, and regenerative medicine.

Further control over release kinetics is achieved by modulating the molecular weight and the nature of functional end-groups in the PLGA polymer chain, as extensively demonstrated in various studies investigating PLGA-based drug delivery systems.^25^ Higher molecular weight polymers typically exhibit slower degradation due to their longer chain lengths and reduced chain-end availability for hydrolytic attack, thereby prolonging drug release. Similarly, the presence of ester-terminated end groups slows hydrolysis compared to acid-terminated variants, which accelerate degradation through autocatalysis.^26^ This tunability provides a powerful means of customizing drug release profiles to meet the pharmacokinetic requirements of specific therapeutic applications. In the work of Allahyari *et al.* (2016), poly(d, l-lactide-*co*-glycolide) (PLGA) particles were highlighted for their biocompatibility and capacity to provide controlled antigen release for vaccine applications.^25^ The authors emphasized that the release behavior of encapsulated proteins and peptides is dictated by both intrinsic polymer features—such as molecular weight and end-group chemistry—and extrinsic formulation variables. These parameters collectively govern the degradation profile of the particles and the diffusion characteristics of the embedded therapeutic agents. By leveraging these molecular design aspects, PLGA systems can be optimized to sustain antigen exposure, which is critical for mounting effective immune responses. Particularly notable is the co-delivery of immunostimulatory molecules and antigens within PLGA, which not only amplifies immunogenicity by targeting dendritic and natural killer cells but also minimizes systemic adverse effects. Moreover, targeting capabilities were improved through functionalization strategies such as surface-decoration to favor M-cell uptake in oral vaccines, illustrating how precise polymer modifications can steer the release behavior and biodistribution of PLGA-based therapeutics.

**Fig. 1:**
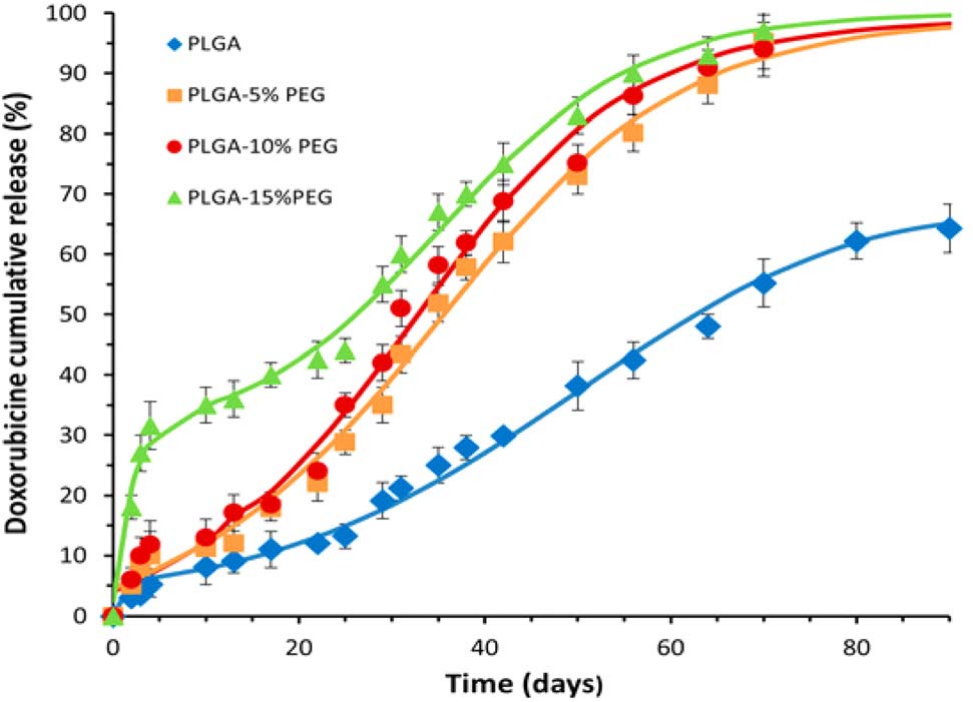
Cumulative ACM release from MP of PLGA-PEG with 5%, 10% and 15% of PEG.^26^ This figure is reused from an article published by MDPI under the Creative Commons CC BY license. No special permission is required for reuse, provided that the original source is properly cited.

In a more recent investigation, Costal *et al.* (2025) explored the influence of polyethylene glycol (PEG) incorporation into PLGA matrices on both degradation kinetics and drug release dynamics.^26^ Their study employed PLGA-PEG microspheres loaded with clacinomycin A (ACM), prepared with varying PEG contents (0%, 5%, 10%, and 15%). Although PEG inclusion had minimal effect on drug loading efficiency and microsphere size, it markedly altered the release behavior by accelerating both matrix degradation and drug liberation. As shown in Fig. 1, cumulative drug release reached over 95% within 70 days for PEG-containing microspheres, while non-PEG PLGA microspheres plateaued at approximately 50%. This stark contrast in release profiles underscores the role of PEG as a modulator of hydrophilicity and chain mobility, thereby facilitating faster water uptake, matrix swelling, and degradation. Importantly, the study observed a triphasic release pattern—characterized by an initial burst (notably 40% for PLGA-15% PEG *vs.* 5% for unmodified PLGA), a diffusion-controlled second phase, and an erosion-driven third phase. The transition into the third phase, where polymer erosion dominates, was found to be strongly dependent on the PEG content, with higher PEG levels precipitating earlier onset of this phase. This temporal shift correlated with the polymer's molecular weight reduction to a critical threshold (∼20 000 Da), occurring progressively earlier in formulations with greater PEG proportions (*e.g.*, 2.5 days for PLGA-15% PEG compared to 34 days for standard PLGA). Such findings reinforce the principle that modifying polymer molecular weight and terminal functionalities can effectively tailor the drug release timeline, thereby providing a strategic handle for designing precision nanomedicine platforms.

In practical applications, drug release from poly(lactic-*co*-glycolic acid) (PLGA) matrices exhibits a well-characterized triphasic release profile that reflects the complex interplay between diffusion, degradation, and erosion mechanisms. The first phase—commonly referred to as the initial burst—is attributed to the rapid diffusion of drug molecules that are either loosely bound to or embedded near the polymer surface. This phase is heavily influenced by the drug's solubility, distribution within the matrix, and the surface area-to-volume ratio of the delivery system.^27,28^

Following the burst, a second, more gradual phase emerges, typically governed by diffusion through a hydrated but increasingly dense polymer matrix. During this stage, the hydrolysis of ester linkages proceeds at a moderate pace, and drug molecules entrapped within the inner regions of the PLGA matrix begin to migrate outward. However, drug mobility remains restricted due to the semi-intact nature of the polymer network, resulting in a controlled, diffusion-limited release rate.^28,29^

The third and final phase is characterized by a marked acceleration in drug release, often described as erosion driven. This occurs when the molecular weight of the degrading polymer drops below a critical threshold, leading to a substantial loss of matrix integrity. Water infiltration increases significantly, triggering bulk degradation and collapse of the polymer structure. As the PLGA continues to break down into its monomeric components—lactic and glycolic acid—these degradation products accumulate within the matrix, creating an increasingly acidic microenvironment that further catalyzes the degradation process. This autocatalytic feedback loop, especially prevalent in the matrix core, contributes to an unpredictable but rapid release of the remaining drug payload.^11,29^

Ford *et al.* (2011) offer a comprehensive modelling framework that captures the dynamics of these sequential release phases.^24^ Their analysis highlights the significant roles played by internal variables such as matrix porosity, particle size, polymer molecular weight, and microclimate pH in dictating the rate and extent of each phase. Particularly, their work underscores how localized autocatalysis—caused by the accumulation of acidic degradation byproducts—can lead to heterogeneous degradation patterns, thereby accelerating the onset of the erosion phase in a spatially non-uniform manner. These findings emphasize the need for careful control over formulation parameters to tailor PLGA-based delivery systems for specific therapeutic windows and ensure consistent performance across *in vitro* and *in vivo* environments.

In the context of inner ear applications, these release dynamics are especially valuable, given the anatomical barriers, delicate cellular structures, and the need for sustained therapeutic exposure within the cochlear and vestibular compartments. The ability of PLGA to orchestrate a controlled, multi-phase release allows for precise modulation of drug availability—minimizing the risks associated with peak-dose toxicity while maintaining therapeutically relevant concentrations over extended periods. This is particularly critical for inner ear disorders, where abrupt or excessive exposure to bioactive agents can exacerbate cochlear damage or disrupt the delicate ionic balance essential for auditory transduction. Furthermore, the progressive erosion of PLGA enables localized drug deposition in hard-to-reach niches within the cochlea, enhancing the delivery of neuroprotective, anti-inflammatory, or regenerative compounds directly to target sites. These controlled kinetics, combined with PLGA's biocompatibility and ability to degrade into metabolizable byproducts, underscore its unique suitability for long-acting, minimally invasive inner ear therapeutics. The study by Wang *et al.* (2023) offers a compelling insight into the application of controlled and sustained drug delivery systems for inner ear disorders, particularly those induced by cisplatin (CP)—a chemotherapeutic agent known for its ototoxic side effects.^17^ In this context, the study underscores the therapeutic advantages of local administration of microcrystal-based formulations over conventional systemic drug delivery, especially when attempting to circumvent physiological barriers such as the blood-labyrinth barrier. This barrier often limits drug permeation into the cochlear fluids, making localized approaches especially attractive for targeting inner ear pathologies.

In the study, non-spherical microcrystals (MCs) of dexamethasone (DEX) and lipoic acid (LA) were prepared without requiring complex chemical modification, highlighting a simple yet scalable formulation approach. These microcrystals were thoroughly characterized for morphology, stability, injectability, and dissolution behavior, as well as their distribution along the round window membrane (RWM)—a critical route for transmembrane diffusion into the inner ear.

The authors administered DEX MCs, LA MCs, and a combination of both into the middle ear of guinea pigs *via* intratympanic injection, a minimally invasive delivery method commonly used in both preclinical and clinical otologic therapies. Importantly, the combination group—co-delivery of DEX and LA MCs—showed a synergistic effect, not only enhancing the absorption of DEX but also providing superior otoprotection by preserving cochlear hair cell integrity in the face of cisplatin-induced injury. This synergism can be attributed to the complementary mechanisms of action of the two agents: DEX acts primarily as an anti-inflammatory corticosteroid, while LA serves as a potent antioxidant. Their concurrent and sustained release ensures prolonged therapeutic levels in the cochlear milieu, mitigating the inflammatory and oxidative stress responses that underlie CP ototoxicity.

Furthermore, the biocompatibility profile of the formulations was favorable, as no significant inflammatory changes or tissue damage were observed in cochlear tissues following treatment. This is particularly critical in the context of inner ear applications, where even minor inflammatory insults can exacerbate sensorineural hearing loss.

The release dynamics demonstrated in this study are especially relevant to inner ear applications because of the limited regenerative capacity of cochlear hair cells, making early and sustained intervention pivotal. By maintaining localized therapeutic concentrations over time, such microcrystal-based systems potentially reduce the need for repeated dosing, thereby improving patient compliance and reducing systemic side effects. The findings of Wang *et al.* (2023) not only validate the efficacy and safety of local, sustained delivery of DEX and LA microcrystals for cisplatin-induced hearing loss but also reinforce the broader utility of controlled-release systems in inner ear therapy.^17^ Their study sets a strong precedent for the integration of anti-inflammatory and antioxidant co-delivery strategies, especially those leveraging biodegradable and biocompatible platforms, to enhance therapeutic outcomes in otologic disorders.

Hydrogel-based formulations further enhance the therapeutic performance of PLGA systems by improving local retention and offering additional modulation of release kinetics. In a notable example, Kim *et al.* (2021) developed a thermosensitive triblock copolymer hydrogel composed of PLGA–PEG–PLGA, into which dexamethasone-loaded nanoparticles were incorporated.^30^ This hydrogel remained in a sol state at room temperature but transitioned to a gel at physiological temperatures, making it ideal for intratympanic administration. Upon injection into the middle ear of mice, the formulation demonstrated a sustained release of dexamethasone over more than seven days, while preserving normal auditory function and showing no histological evidence of tissue inflammation or cochlear damage. This dual-level delivery strategy—leveraging both the nanoparticulate encapsulation and the thermoresponsive hydrogel matrix—effectively addresses two major challenges in otologic drug delivery: short residence time due to middle ear clearance mechanisms, and the need for sustained, controlled release to achieve otoprotection without repeated administration.

Taken together, these findings emphasize the adaptability of PLGA-based systems to meet the specific demands of inner ear therapy. By tuning polymer composition, molecular weight, terminal group functionality, and integrating PLGA into advanced delivery platforms such as injectable hydrogels, it is possible to engineer formulations that offer precise, predictable, and long-lasting therapeutic profiles. Importantly, the demonstrated *in vivo* safety, compatibility with delicate auditory structures, and flexibility in carrying both small molecules and biologics position PLGA as one of the most promising materials for translational application in otologic medicine—whether for the delivery of anti-inflammatory corticosteroids, reactive oxygen species scavengers, or regenerative peptides and gene vectors.

### 2.3 Surface functionalization

Surface functionalization plays a pivotal role in enhancing the effectiveness of PLGA-based nanoparticles for inner ear therapies. By engineering the nanoparticle surface, researchers have successfully improved traversal of the round window membrane (RWM), increased uptake by target cochlear cells, and facilitated escape from endosomes—key processes for delivering therapies across this highly selective barrier.^14^

One widely adopted strategy is PEGylation, where the nanoparticle surface is grafted with polyethylene glycol (PEG). This hydrophilic “stealth” coating reduces protein adsorption, minimizes phagocytic clearance, and importantly, improves diffusion through the extracellular matrix and inner ear fluids.^31^ A particularly effective design integrates PEGylation with ligand conjugation to combine stealth properties with targeting capabilities. Beyond PEGylation, surface functionalization enhances nanoparticle–cell interactions by allowing conjugation with peptides, antibodies, or aptamers that recognize specific receptors on cochlear hair cells or spiral ganglion neurons. Such functional groups facilitate receptor-mediated endocytosis, improving intracellular delivery and therapeutic retention. Moreover, surface modification can be tailored to modulate nanoparticle charge and hydrophobicity, optimizing membrane affinity and drug release kinetics. These physicochemical enhancements collectively improve biocompatibility, stability, and site-specific drug accumulation, thereby amplifying the therapeutic efficacy of PLGA nanoparticles in otoprotection and inner ear regeneration.^32,33^ Dindelegan *et al.* (2022) comprehensively reviewed such approaches, highlighting systems that use PEGylated PLGA or PLA backbones functionalized with peptides like TET1, which targets spiral ganglion neurons, or A666, specific to outer hair cells.^31^

A standout example of ligand-mediated targeting is the A666-conjugated PEG–PLA nanoparticle, engineered by Wang *et al.* (2018). These nanoparticles carry dexamethasone and are decorated with A666 to specifically bind to prestin, a motor protein located on outer hair cells (OHCs).^32^ The nanoparticles remained ∼80 nm in size and demonstrated stable physical properties even after conjugation. Confocal microscopy revealed that, within two hours of intratympanic administration, A666-tagged nanoparticles were significantly more concentrated in OHC cytoplasm than untargeted particles. Functional studies showed that this targeted delivery markedly improved hair cell survival and preserved hearing after cisplatin-induced damage—outcomes unattainable with non-targeted nanoparticles.

Beyond peptide conjugation, chitosan coating has emerged as another powerful surface modification. Chitosan's positively charged and mucoadhesive properties enable prolonged retention on the RWM and enhance cellular uptake. Saber *et al.* (2010) demonstrated in guinea pigs that glycosylated chitosan nanoparticles loaded with neomycin could cross the RWM safely, without inducing structural or inflammatory damage in cochlear tissues. This early work laid the foundation for modern inner ear nanotherapeutics.^33^

Building on this, Chen *et al.* (2022) developed curcumin-loaded chitosan-coated PLGA nanoparticles (CUR-CS/PLGA). These were evaluated using HEI-OC1 hair cell models and in guinea pig cochleae, showing significantly enhanced cochlear retention and cellular uptake compared to both uncoated PLGA particles and free curcumin.^34^ Crucially, the formulation led to robust otoprotection—curbing apoptosis and oxidative stress—thus confirming that chitosan functionalization can substantially boost the therapeutic profile of antioxidant agents targeting the inner ear. The combination of these surface modifications illustrates a powerful design paradigm: PEGylation offers stealth and diffusion benefits, ligand conjugation ensures targeted delivery to specific cochlear cell types, and chitosan coating enhances membrane adherence and internalization. Together, these strategies enable PLGA-based platforms to deliver a wide range of therapeutic agents—steroids, antioxidants, neurotrophins, or gene editing tools—with enhanced precision and efficacy. Such functionalized nanoparticles not only cross the RWM more efficiently but also localize within the appropriate cell types and deliver their payload intracellularly, making them promising candidates for clinical translation in otologic medicine.

Sensorineural hearing loss (SNHL) remains a global health concern, particularly within aging populations, where the degeneration of cochlear hair cells and spiral ganglion neurons (SGNs) underlies most cases. Rathnam *et al.* (2019) provide a compelling overview of how aminoglycoside antibiotics, acoustic trauma, and age-related degeneration collectively affect cochlear structures, contributing to hearing impairment in approximately 15% of the adult American population—around 36 million individuals.^35^ With life expectancy on the rise, the burden of SNHL is projected to increase substantially. Current treatment modalities, such as hearing aids and cochlear implants, offer only partial rehabilitation. Hearing aids require functional hair cells to amplify sound, and cochlear implants are ineffective without healthy SGNs to transmit neural signals. Hence, preserving cochlear cell integrity or regenerating lost cells is imperative. However, one of the critical barriers to therapeutic intervention is the difficulty of drug delivery to the inner ear. The cochlea's anatomical seclusion, coupled with the presence of the blood-labyrinth barrier, limits systemic drug availability and necessitates local delivery methods.

Rathnam *et al.* (2019) emphasize that novel biomaterials, particularly hydrogels, are at the forefront of solving these challenges.^35^ Hydrogels, defined as hydrophilic polymeric networks capable of swelling and retaining large amounts of water, serve as excellent drug delivery platforms due to their tunable mechanical and degradation properties, high drug loading potential, and biocompatibility. When administered intratympanically, hydrogels can gel near the round window membrane (RWM), thereby forming a depot for sustained local drug release.

**Fig. 2:**
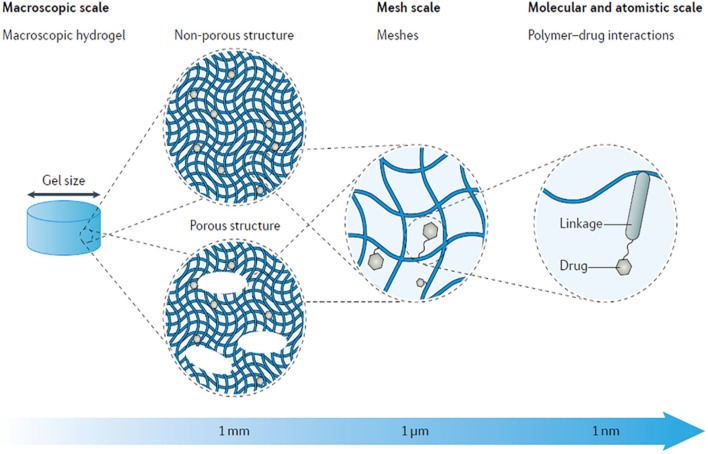
The multi-scale tunability of hydrogels for drug delivery applications, showcasing the versatility of drug delivery properties based on material characteristics.^35^ Copyright © 2019 Rathnam, Chueng, Ying, Lee and Kwan. This article is distributed under the terms of the Creative Commons Attribution (CC BY) License. Reuse of this figure is permitted provided that the original authors and copyright holders are properly credited and the original publication is cited in accordance with academic practice. No use is permitted that does not comply with these terms.

One of the notable advantages of hydrogel-based systems is their capacity to prolong drug residence time near the cochlear interface. This is particularly important because achieving therapeutic drug concentrations in the perilymph over a sustained period significantly increases treatment efficacy. The increased viscosity of these systems enhances retention and improves diffusion kinetics across the RWM, addressing one of the primary limitations of simple intratympanic injections. Rathnam *et al.* (2019) highlight that hydrogels can be derived from either natural or synthetic sources, each offering unique benefits.^35^ Natural hydrogels—such as alginate, gelatin, and hyaluronic acid—are favored for their inherent biocompatibility and minimal immunogenicity. On the other hand, synthetic hydrogels—such as those based on polyethylene glycol (PEG) or poly(lactic-*co*-glycolic acid) (PLGA)—provide superior control over mechanical strength, degradation rate, and functionalization capacity. Fig. 2 illustrates the wide array of hydrogel types classified based on their chemical composition and physical properties underscoring the customizable nature of these delivery platforms.

Of particular relevance to the discussion on ligand conjugation and cochlear cell specificity, Rathnam *et al.* (2019) contextualize how surface modifications can further enhance hydrogel and nanoparticle performance.^35^ By conjugating ligands such as cell-penetrating peptides (CPPs) or antibodies, drug carriers can selectively bind to and be internalized by specific cochlear cell types, including hair cells and SGNs. This ligand-mediated targeting is crucial not only for bypassing anatomical barriers such as the RWM but also for improving intracellular delivery, minimizing off-target effects, and enhancing therapeutic outcomes. The study by Rathnam *et al.* (2019) underscores that the integration of hydrogel-based carriers with surface-functionalized nanostructures provides a synergistic platform for sustained, localized, and targeted drug delivery to the inner ear.^35^ The convergence of bioresponsive materials, ligand-guided targeting, and microsurgical advances presents a transformative potential for addressing sensorineural hearing loss at the cellular level.

Together, these surface functionalization techniques—such as PEGylation, chitosan coating, the use of hydrophilic surfactants, and ligand conjugation—play a pivotal role in optimizing the performance of PLGA-based nanocarriers for inner ear drug delivery. PEGylation improves systemic circulation time and minimizes immune clearance, while chitosan coating offers mucoadhesive properties and facilitates permeation across epithelial barriers. Hydrophilic surfactants enhance nanoparticle dispersion and stability in the perilymphatic environment, and ligand conjugation (*e.g.*, peptides targeting outer hair cells or prestin receptors) promotes selective binding and internalization by specific cochlear cells. Collectively, these modifications not only increase the efficiency of nanoparticle penetration through the round window membrane but also enhance cellular uptake within the cochlear microenvironment. As such, these strategies are indispensable in the development of next-generation PLGA nanocarriers, ensuring targeted, safe, and efficient therapeutic delivery to protect sensory hair cells, promote neuronal survival, or facilitate inner ear regeneration—ultimately advancing the clinical translation of nanomedicine for hearing restoration.

### 2.4 Size and zeta potential

Recent research efforts underscore the critical importance of optimizing nanoparticle size and surface characteristics to enhance the efficiency of intratympanic drug delivery to the inner ear.^15^ The structural and physiological barriers posed by the round window membrane (RWM) necessitate precise engineering of nanoparticulate systems to ensure adequate permeability and therapeutic payload transport.^36^ Among the most influential parameters, particle size plays a pivotal role in determining not only membrane penetration but also biodistribution within the cochlear fluids.^37^ Studies have consistently demonstrated that nanoparticles with diameters at or below 300 nm—particularly those closer to 200 nm—are more likely to traverse the RWM effectively,^38^ as their reduced size facilitates paracellular transport and minimizes steric hindrance.

Additionally, smaller nanoparticles tend to exhibit lower rates of mucociliary clearance in the middle ear cavity, thereby extending their residence time and enhancing the probability of cochlear entry.^39^ Moreover, such nano-scaled systems are less prone to aggregation within the cochlear perilymph, ensuring more uniform drug dispersion and sustained therapeutic activity.^40^ Taken together, these findings reinforce the notion that careful manipulation of nanoparticle dimensions and surface properties is essential for overcoming the anatomic and fluidic challenges of inner ear drug delivery. Among such efforts, the study by George *et al.* (2019) offers strong preclinical evidence demonstrating that solid lipid nanoparticles (SLNs)—which are not PLGA-based—sized between 100 and 200 nm exhibit superior *trans*-RWM transport and efficient uptake by target cochlear cells.^41^ These findings substantiate the widely held notion that sub-200 nm particles are especially favourable for crossing biological membranes like the RWM, owing to their enhanced diffusivity and reduced steric hindrance. Although SLNs differ compositionally from PLGA nanoparticles, the observed size-dependent behaviour provides valuable comparative insight into the importance of nanoscale dimensions for optimizing inner ear drug delivery.

Expanding on this size-dependent behaviour, Cai *et al.* (2017) investigated deeper into the synergy between nanoparticle size, surface functionality, and penetration enhancers.^42^ Their investigation demonstrated that PLGA nanoparticles in the 100–300 nm range could traverse the RWM with different efficiencies depending on surface modifications. Chitosan-coated (positively charged) nanoparticles and hydrophilic surfactant-modified PLGA nanoparticles (*e.g.*, using Poloxamer 407) exhibited markedly enhanced cochlear entry. Notably, the combination of surface hydrophilicity and cationic charge allowed for better membrane interaction and deeper distribution within cochlear tissues. The study further employed low molecular weight protamine (LMWP) as a cell-penetrating peptide (CPP), which significantly enhanced cellular uptake and facilitated delivery to sensitive structures like the organ of Corti and the stria vascularis without inflicting damage, again demonstrating the central role of nano-scale formulation coupled with appropriate surface engineering.

Complementing these experimental findings, Mäder *et al.* (2018) presented a comprehensive review that synthesized recent developments in inner ear drug delivery, highlighting the influence of size, shape, and physicochemical properties on nanoparticle diffusion and release kinetics.^43^ The review underscored that drug carriers with nanometric dimensions—notably under 200–300 nm—offered more predictable and tunable release behaviors due to their higher surface area-to-volume ratio and interaction with biological barriers. Furthermore, the authors called attention to the necessity of interdisciplinary collaboration in designing robust nanoformulations that can reliably navigate the unique challenges of inner ear delivery, including anatomical constraints, fluid dynamics, and biological reactivity.

In parallel, Wang *et al.* (2018) provided compelling evidence for the synergistic effects of particle size and targeted surface conjugation in augmenting drug specificity.^32^ In their study, phospholipid-based nanoparticles approximately 200 nm in size were conjugated with the A666 peptide and engineered to bear a moderate cationic charge (zeta potential of approximately +25 mV). This dual functionalization strategy enabled selective targeting of prestin-expressing outer hair cells, a key cellular component affected in sensorineural hearing loss. The enhanced penetration and targeted delivery achieved in this study underscore how precise modulation of both size and surface chemistry can translate into improved therapeutic precision and efficacy.

Additionally, surface charge and hydrophilicity play indispensable roles in mediating nanoparticle interaction with the cochlear membranes and their subsequent distribution in the perilymph. Aibani *et al.* (2021) demonstrated that chitosan-based nanoparticles (not PLGA-based), particularly those possessing a positive surface charge, significantly increased adhesion to the round window membrane (RWM) due to enhanced electrostatic interactions with its negatively charged extracellular matrix.^44^ This improved both nanoparticle retention and cellular uptake. However, the authors also cautioned that excessively high surface charge could result in non-specific binding, uncontrolled distribution, and potential cytotoxicity. This observation highlights an important design consideration—the need to balance surface charge for optimal efficacy and biocompatibility in nanoparticle-mediated inner ear delivery systems, even though the findings were based on non-PLGA formulations.

To address such challenges, studies like those by Chen *et al.* (2022)^34^ and Saber *et al.* (2010)^33^ propose the integration of hydrophilic surface modifications, such as PEGylation or surfactant coatings, into nanoparticle platforms. These modifications confer several advantages: they stabilize the nanoparticles in the cochlear fluids, prevent premature aggregation, and enhance uniform dispersion throughout the inner ear structures. Such hydrophilic coatings also reduce protein adsorption and immune recognition, thereby improving biocompatibility and prolonging nanoparticle circulation within the targeted cochlear compartments.

Altogether, these studies converge on a common conclusion: that nanoparticles designed for inner ear applications—whether for otoprotection, regeneration, or inflammation control—must be finely tuned in both size (preferably ≤200 nm) and surface properties. Charge balance, hydrophilicity, and biocompatibility are not merely auxiliary traits but essential components in ensuring safe and effective drug delivery. Through meticulous nanoparticle engineering, it becomes increasingly feasible to deliver therapeutic agents precisely and safely to delicate auditory structures, potentially revolutionizing the treatment landscape for sensorineural hearing loss and related otologic disorders.

## 3. Routes of administration and cochlear targeting

### 3.1 Delivery and round window membrane penetration

Intratympanic (IT) delivery has emerged as a cornerstone strategy for local drug administration to the inner ear, offering distinct advantages over systemic routes, particularly in bypassing physiological barriers such as the blood-labyrinth barrier, which restricts drug passage from systemic circulation into the cochlea. The underlying anatomical framework of the ear supports this targeted delivery approach. The ear comprises three major compartments: the external ear, the middle ear, and the inner ear. The middle ear, located between the tympanic membrane and the oval and round windows, houses the ossicles and serves as a critical chamber for the IT administration of therapeutics.

At the interface between the middle and inner ear lies the round window membrane (RWM), a delicate trilaminar structure that functions as a semi-permeable biological barrier. It facilitates selective diffusion of molecules from the middle ear into the perilymph of the scala tympani, one of the three fluid-filled compartments of the cochlea. This anatomical positioning makes the RWM an ideal gateway for drug entry into the cochlea following IT injection. The RWM consists of an outer epithelium facing the middle ear, a central connective tissue layer, and an inner epithelium lining the scala tympani. Its semi-permeable nature allows for passive diffusion of small molecules and, with the aid of formulation strategies like nanoparticles, even larger macromolecules or sustained-release systems.

The clinical relevance of IT delivery stems from its ability to directly target cochlear structures such as inner and outer hair cells, the stria vascularis, and spiral ganglion neurons—sites critically involved in hearing and commonly affected in sensorineural hearing loss. This mode of delivery allows for higher local drug concentrations and reduces systemic exposure and toxicity. Moreover, IT injection is minimally invasive and can be performed repeatedly in outpatient settings, enhancing patient compliance.

Given the narrow and delicate structure of the cochlea, achieving therapeutic drug levels *via* systemic routes is highly inefficient and often associated with off-target effects. Thus, IT delivery through the RWM represents a rational and focused method to overcome the pharmacokinetic limitations of inner ear therapies. It is especially valuable for chronic conditions requiring sustained drug presence in the cochlea, where biodegradable nanoparticles like PLGA—often modified with permeation enhancers such as chitosan or surfactants—can further augment drug passage across the RWM and improve therapeutic retention.

For example, Xiao *et al.* (2021) emphasize the critical barriers that impede drug delivery *via* systemic routes, such as the blood-labyrinth barrier and the limited vascularization of the inner ear.^45^ They highlight that IT injection not only circumvents these challenges but also ensures drug deposition near the cochlear and vestibular compartments, facilitating diffusion through either the round or oval windows. This localized administration enhances drug concentration at the target site while reducing systemic side effects. Their review draws attention to the evolving role of nanoparticle-based drug carriers in improving retention times and achieving targeted delivery to hair cells or synaptic zones. These systems are designed to interact favorably with the RWM, allowing for controlled and sustained drug permeation into the inner ear structures.

Moatti *et al.* (2023) contribute a translational perspective by addressing the physical limitations of the RWM as a diffusion barrier.^46^ Their study underscores the fact that only a limited subset of molecules can naturally penetrate this membrane, necessitating innovative strategies to improve permeability. To this end, they developed an ex vivo porcine RWM model, structurally analogous to the human RWM, to evaluate drug diffusion and retention over time. This model enables precise measurement of molecular transport across the RWM, serving as a valuable tool for screening nanoparticle formulations and optimizing IT drug delivery strategies. Their findings reinforce the concept that IT injection, though non-invasive compared to surgical options like cochleostomy, still requires careful formulation design to overcome the selective permeability of the RWM.

Together, these studies affirm that intratympanic administration is not only the most clinically relevant route for delivering therapeutics to the inner ear, but also a technically dynamic one—requiring integration of anatomical insights, pharmacokinetic optimization, and advanced biomaterials engineering to ensure efficient RWM penetration and therapeutic efficacy.

For example, in this context, the use of poly(lactic-*co*-glycolic acid) (PLGA) nanoparticles modified with chitosan or surfactant coatings has emerged as a highly promising approach to enhance permeability and retention of therapeutics in cochlear tissues.^47,48^ The choice of chitosan—a cationic, mucoadhesive polysaccharide—leverages its ability to transiently open tight junctions and interact electrostatically with negatively charged mucosal surfaces of the RWM, thereby facilitating nanoparticle uptake and transmembrane transport.^48,49^ Chitosan-coated PLGA NPs have been shown to significantly improve drug residence time in the perilymph and offer sustained therapeutic exposure, which is especially beneficial in managing progressive cochlear injuries.^50^

Similarly, surfactant-modified PLGA NPs, such as those coated with poloxamers, Tween-80, or sodium taurocholate, exhibit enhanced membrane fluidity and promote the paracellular and transcellular penetration of the nanoparticulate payload.^51,52^ These surfactants can modulate the lipid bilayer of the RWM, increasing permeability without causing structural damage. Studies using fluorescently labelled PLGA-surfactant NPs demonstrate deeper cochlear diffusion, with higher accumulation around target regions like the organ of Corti and spiral ganglion, as compared to unmodified nanoparticles.^53,54^

Several preclinical studies have validated these enhancements. For instance, Wang *et al.* (2018) reported that PLGA nanoparticles modified with Tween-80 effectively delivered antioxidants into inner ear tissues, reducing oxidative stress in a model of cisplatin-induced ototoxicity.^32^ These coatings not only facilitate drug delivery but also protect labile compounds from degradation in the middle ear environment.

Altogether, these advancements underline a synergistic relationship between anatomical understanding, delivery route optimization, and smart biomaterial engineering. By leveraging the advantages of IT administration and enhancing nanoparticle formulations with chitosan or surfactants, researchers have moved closer to overcoming the formidable challenge of inner ear drug delivery—enabling higher therapeutic precision, longer residence times, and reduced dosing frequency. These technologies offer strong translational potential for treating a variety of otologic disorders, including sensorineural hearing loss, tinnitus, and drug-induced ototoxicity.

In the study by Kim *et al.* (2021), the potential of PLGA nanoparticles (NPs) with surfactant coatings, specifically thermosensitive poloxamer-based gels, to enhance permeability and retention within the cochlea was evaluated through a series of *in vitro* and *in vivo* assays.^30^ The investigators formulated dexamethasone (DEX)-loaded PLGA nanoparticles (DEX-NPs), which were subsequently embedded in a poloxamer gel matrix to yield a thermoresponsive DEX-NP-gel system. The NPs exhibited an average particle size of 150.0 ± 3.2 nm, a zeta potential of −18.7 ± 0.6 mV, and an encapsulation efficiency of approximately 64.4%, ensuring appropriate physicochemical properties for intratympanic (IT) delivery.

**Fig. 3:**
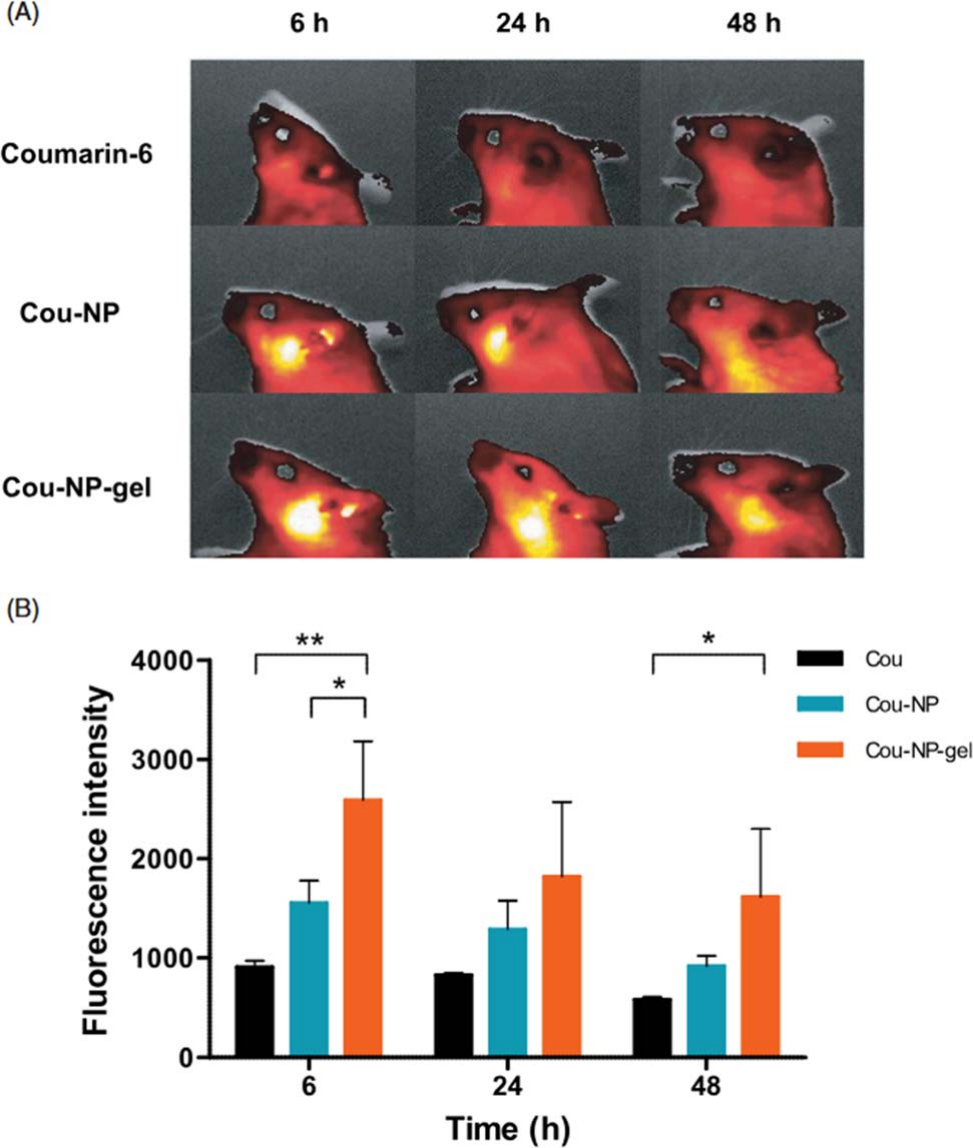
*In vivo* fluorescence imaging and quantification. (A) Representative fluorescence images and (B) corresponding fluorescence intensity of free coumarin-6 (Cou), coumarin-6-loaded nanoparticles (Cou-NP), and coumarin-6-loaded nanoparticles incorporated in thermosensitive gel (Cou-NP-gel) following administration. Data are presented as mean ± standard deviation (*n* = 3). Statistical analysis was performed using one-way ANOVA followed by Tukey's post hoc test; Cou-NP-gel showed significantly higher fluorescence intensity compared to both Cou and Cou-NP (*p < 0.05, **p < 0.01).^30^ This figure is published under the Creative Commons CC BY-NC License, which permits non-commercial use, distribution, and reproduction in any medium, provided the original work is properly cited.

The key innovation in this study was the application of a surfactant-coated PLGA system using poloxamer thermogels to prolong the formulation's residence time in the middle ear. This was clearly demonstrated in the *in vivo* fluorescence imaging experiment using coumarin-6 as a fluorescent marker. After IT administration, Cou-NP-gel showed prolonged retention of fluorescence in the middle ear, persisting up to 48 hours, while the solution (Cou) was largely cleared by 6 hours. Fluorescence quantification revealed that Cou-NP-gel retained approximately 60% of its initial intensity at 48 hours, which was about three times greater than Cou alone, confirming enhanced local retention and slower clearance (Fig. 3).

**Fig. 4:**
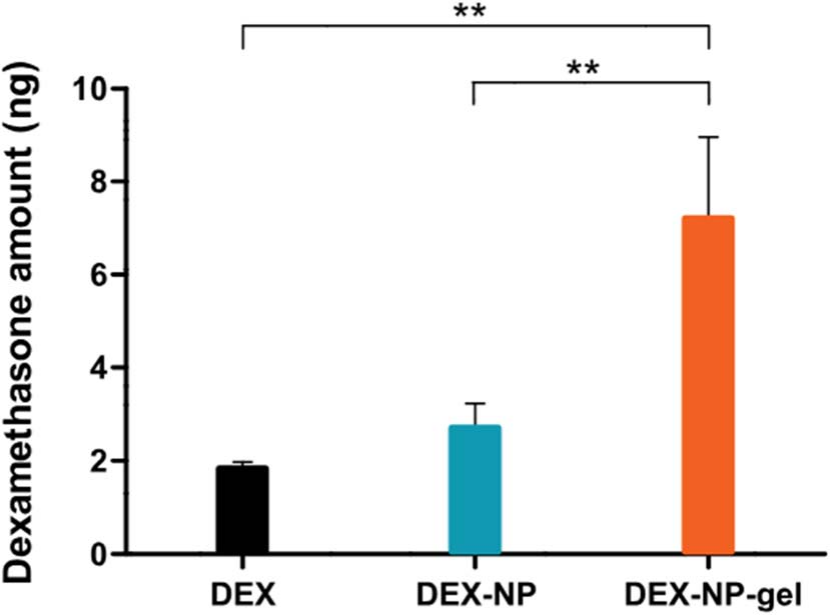
Residual concentration of dexamethasone (DEX) in cochlear tissues 24 hours post-administration. The amount of DEX remaining in the cochlea was measured 24 hours after treatment with free DEX solution, DEX-loaded nanoparticles (DEX-NP), and DEX-loaded nanoparticles incorporated into thermosensitive gel (DEX-NP-gel). Data are presented as mean ± standard deviation (*n* = 3). Statistical analysis using one-way ANOVA followed by Tukey's post hoc test revealed that DEX-NP-gel retained significantly higher DEX levels in the cochlea compared to both DEX and DEX-NP (**p < 0.01).^30^ This figure is published under the Creative Commons CC BY-NC License, which permits non-commercial use, distribution, and reproduction in any medium, provided the original work is properly cited.

To assess cochlear penetration, a biodistribution study was conducted in which DEX levels in the cochlea were measured following IT administration of different formulations. Results showed that DEX-NP-gel delivered 7.20 ± 1.76 ng of DEX to the cochlea—nearly four times higher than the DEX solution and more than 2.5 times higher than uncoated DEX-NPs (Fig. 4). This increased delivery was attributed to the gel's sustained-release profile and extended contact with the round window membrane (RWM), enhancing permeability. These findings directly support the assertion that surfactant (poloxamer) coating on PLGA NPs improves both RWM penetration and cochlear retention of therapeutic agents.

**Fig. 5:**
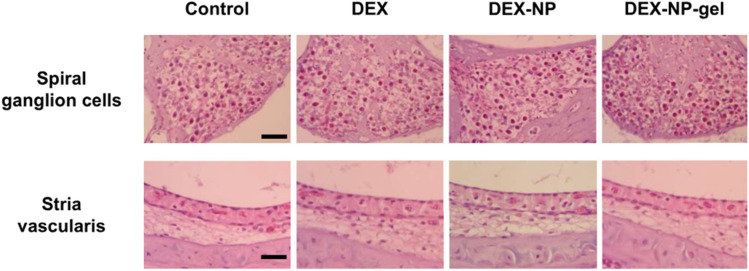
Histological evaluation of the spiral ganglion cells and stria vascularis in the mouse cochlea using H&E staining. No significant morphological alterations were observed in the spiral ganglion cells. Scale bars: 40 µm (spiral ganglion cells) and 20 µm (stria vascularis).^30^ This figure is published under the Creative Commons CC BY-NC License, which permits non-commercial use, distribution, and reproduction in any medium, provided the original work is properly cited.

Importantly, histological evaluations (Fig. 5) of the cochlear structures, particularly the spiral ganglion cells and stria vascularis, revealed no significant morphological changes or inflammatory responses following IT administration of DEX-NP-gels. Despite *in vitro* cytotoxicity noted at 1 mg mL^−1^, no adverse effects were observed *in vivo* even at 4 mg mL^−1^, underscoring the biocompatibility of the PLGA-poloxamer delivery system within the sensitive cochlear environment.

Collectively, the findings from Kim *et al.* (2021) affirm that PLGA NPs modified with surfactant-based gels (such as poloxamer) provide enhanced cochlear drug retention, improved RWM permeability, and safe delivery profiles.^30^ This positions such formulations as promising candidates for targeted and sustained intratympanic therapies for inner ear disorders.

A relevant study by Asal *et al.* (2022) demonstrates how functionalization of chitosan-based nanoparticles with PLGA significantly alters both their surface charge and delivery performance.^55^ In this work, three insulin-loaded nanoparticle systems were prepared—chitosan nanoparticles (ChNps), chitosan-gold nanoparticles (ChAuNps), and chitosan-gold nanoparticles functionalized with PLGA (ChAuNps/PLGA)—using an emulsion solvent diffusion method. Physicochemical characterization revealed distinct morphological and surface charge properties: while ChNps and ChAuNps were highly positively charged (zeta potential +36 to +33 mV), the ChAuNps/PLGA exhibited a negative surface charge (−31 to −26 mV) due to PLGA coating.

The transition from a cationic to anionic surface has critical implications for mucosal and epithelial permeability. Although this study focused on oral insulin delivery in diabetic rats, its findings offer direct translational relevance for cochlear delivery systems. The RWM is negatively charged and selectively permeable; thus, neutral or slightly negative NPs—such as PLGA-coated ChAuNps—are more likely to traverse this barrier without inducing cytotoxicity or triggering inflammatory responses. Moreover, the ChAuNps/PLGA demonstrated superior entrapment efficiency (99 ± 1.2%), pH-responsive release, and enhanced pharmacological activity (blood glucose reduction of 27 ± 5.6%, PA% of 15.8 ± 0.71). These results validate that PLGA functionalization not only facilitates controlled release and biocompatibility but also optimizes the surface electrostatics for effective *trans*-barrier delivery. Although the cochlear context differs from the gastrointestinal tract, the mechanistic insights from this study support the broader concept that PLGA-modified, biocompatible nanocarriers—engineered with tunable surface charge—can be tailored to maximize RWM penetration, prolong residence time in perilymph, and minimize toxicity, all of which are critical for achieving efficient inner ear drug delivery.

Cai *et al.* (2014) provided critical experimental evidence that directly supports the notion that PLGA nanoparticles (NPs), especially those stabilized with surfactants such as polyvinyl alcohol (PVA), exhibit enhanced permeability and retention within cochlear tissues following intratympanic (IT) administration.^56^ Their study demonstrated that PLGA NPs could traverse the round window membrane (RWM)—a critical barrier separating the middle and inner ear—and significantly elevate drug concentrations in the perilymph (PL) compared to corresponding free drug solutions. By loading the NPs with coumarin-6 as a fluorescent probe and with therapeutic agents like salvianolic acid B (Sal B), tanshinone IIA (TS IIA), and total panax notoginsenoside (PNS), the authors systematically investigated drug penetration, bioavailability, and pharmacokinetics following RWM administration in guinea pigs.

**Fig. 6:**
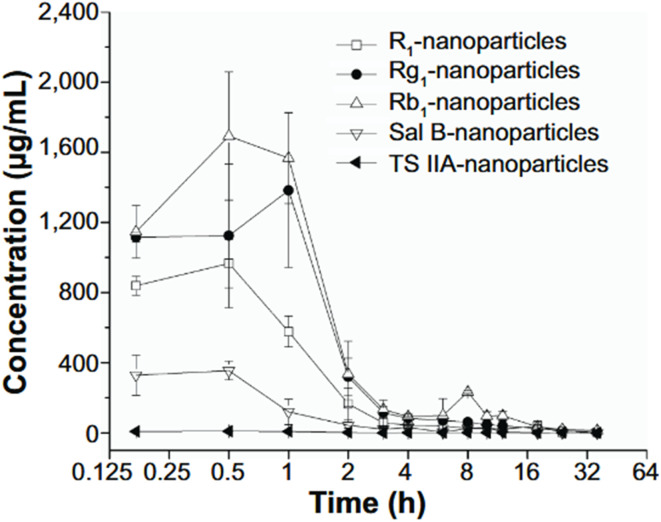
Pl concentration *versus* time curves in guinea pigs after rW administration of Plga NPs loaded with Ts IIa, sal B, and PNs (*n* = 3, mean ± sD). Abbreviations: Pl, perilymph; rW, round window; Plga NPs, poly(d, l-lactide- co-glycolide acid) nanoparticles; Ts IIa, tanshinone IIa; sal B, salvianolic acid B; PNs, panax notoginsenoside; r1, notoginsenoside r1; rg1, ginsenoside rg1; rb1, ginsenoside rb1; sD, standard deviation.^56^ This figure is reproduced under the terms of the Creative Commons CC BY-NC License. It may be used, distributed, and reproduced for non-commercial purposes, provided that the original source is appropriately cited.

Notably, the pharmacokinetic data illustrated striking improvements in drug bioavailability. For instance, the AUC_0_–*t* and Cmax values of coumarin-6 in the PL following NP administration were 4.7- and 10.9-fold higher, respectively, than those from the solution form. For compound drugs like notoginsenoside R1, Rg1, and Rb1, even more dramatic enhancements were reported. Despite being administered at much lower doses *via* the NP formulation, their *C*_max_ values were 14.4-, 10.0-, and 16.7-fold higher, respectively, and AUC_0_–*t* values were significantly elevated as well (4.0-, 3.1-, and 7.1-fold). These enhancements were clearly depicted in Fig. 6 which presented the concentration–time curves of these compounds in the perilymph, revealing prolonged and elevated levels of drug retention compared to the solution form.

**Fig. 7:**
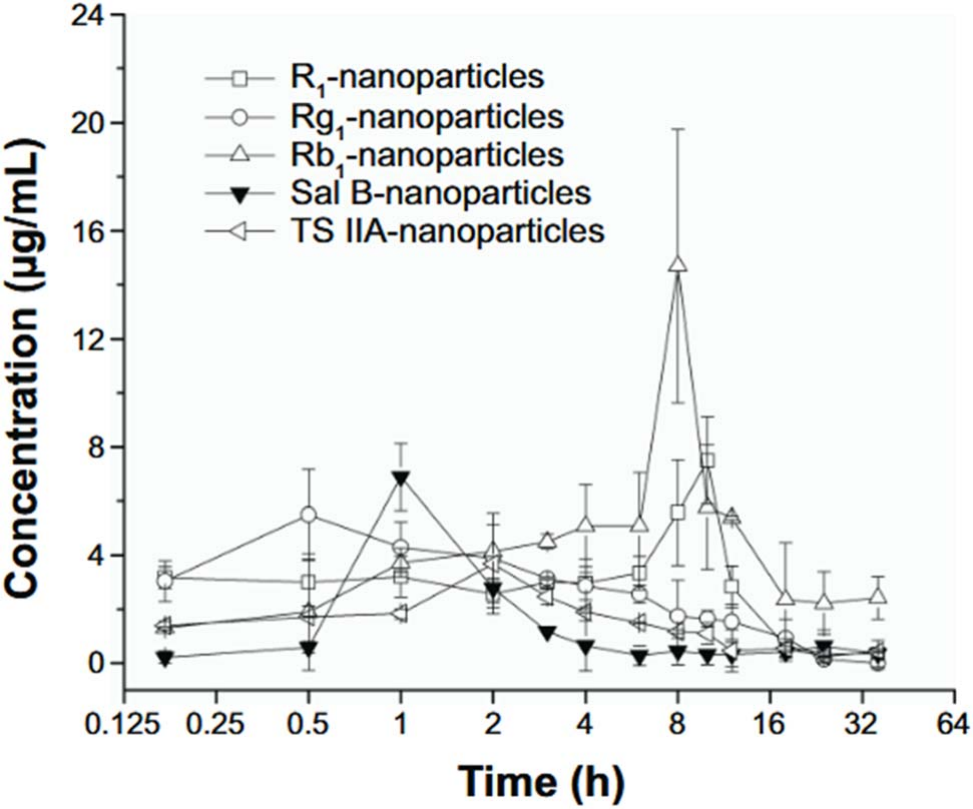
Plasma concentration–time profiles in guinea pigs following round window (rW) administration of PLGA nanoparticles (PLGA NPs) loaded with Tanshinone IIa (Ts IIa), Salvianolic acid B (Sal B), and Panax notoginsenosides (PNs). Data are presented as mean ± standard deviation (SD, *n* = 3). Abbreviations: PL, perilymph; rW, round window; PLGA NPs, poly(d, l-lactide-co-glycolide acid) nanoparticles; Ts IIa, tanshinone IIa; Sal B, salvianolic acid B; PNs, panax notoginsenosides; R1, notoginsenoside R1; Rg1, ginsenoside Rg1; Rb1, ginsenoside Rb1; SD, standard deviation; h, hours.^56^ This figure is reproduced under the terms of the Creative Commons CC BY-NC License. It may be used, distributed, and reproduced for non-commercial purposes, provided that the original source is appropriately cited.

Interestingly, even when the NPs contained significantly reduced doses of Sal B and TS IIA—up to 58.7-fold lower—their pharmacokinetic performance in the PL remained comparable to or slightly lower than the drug solutions, pointing to improved delivery efficiency. The results in Fig. 7 further emphasized the superior retention of drugs within the cochlear compartment when delivered *via* PLGA NPs, a phenomenon attributed to multiple factors including the nanoparticles' small size, bioadhesive potential, and the protective encapsulation of labile drugs.

One major contributor to the enhanced permeability was the ability of the PLGA NPs to traverse the semipermeable RWM, likely through pinocytosis or passive diffusion. Their deposition at the RWM and slow clearance from the middle ear cavity due to the viscous colloidal nature of the formulation allowed prolonged contact with the membrane, improving transport efficiency. Furthermore, the surfactants in the NP suspension, notably PVA, may have facilitated both membrane penetration and systemic distribution, contributing to the observed increases in drug concentration not only in the PL but also in plasma.

Collectively, the findings by Cai *et al.*^42^ substantiate the claim that PLGA NPs, especially when stabilized with surfactants, enhance the permeability of therapeutic agents across the RWM and ensure better retention within cochlear fluids. This highlights their potential as a promising delivery system for treating inner ear disorders through IT injection.

### 3.2 Magnetically guided and ultrasound-enhanced strategies for cochlear targeting: unlocking the potential of PLGA nanoparticles

The targeted delivery of therapeutics to the cochlea remains a formidable challenge in otology, particularly due to the restrictive architecture of the inner ear and the selective permeability of the round window membrane (RWM).^57^ Poly(lactic-*co*-glycolic acid) (PLGA) nanoparticles (NPs) have emerged as promising carriers for drug delivery due to their biocompatibility, tunable degradation rates, and ability to encapsulate a wide range of therapeutics.^58,59^ However, the efficacy of PLGA-based systems largely depends on their capacity to efficiently localize to and penetrate the cochlear perilymph, primarily through the RWM after intratympanic (IT) administration. To address the limitations of passive diffusion, recent advances have focused on magnetic targeting and ultrasound-enhanced strategies, both of which offer external control mechanisms to improve localization, retention, and therapeutic impact of PLGA NPs in inner ear therapy.^58–60^

**Fig. 8:**
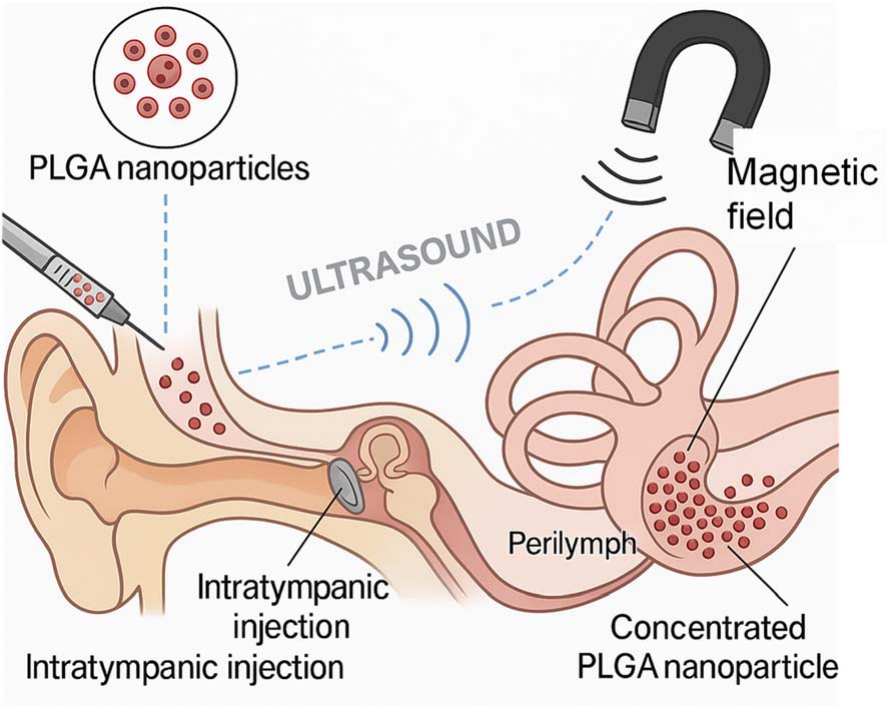
Magnetically guided and ultrasound-enhanced strategies for targeted delivery of PLGA nanoparticles into the cochlea.

Fig. 8 illustrates the conceptual framework for these advanced strategies: magnetically-guided and ultrasound-enhanced approaches are employed to direct PLGA nanoparticles—coated with chitosan or surfactants—across the RWM into the scala tympani. It highlights the key cochlear structures, including the spiral ganglion neurons and sensory hair cells, which are the primary therapeutic targets. The dual-modality enhancement facilitates greater permeability, tissue retention, and controlled site-specific accumulation of therapeutic agents within the inner ear. By coupling these physical targeting modalities with the biochemical properties of PLGA, a synergistic effect is achieved that significantly enhances drug delivery outcomes in cochlear treatment paradigms.

#### 3.2.1 Magnetic targeting using PLGA-based nanoparticles for enhanced cochlear drug delivery

Magnetic targeting represents an advanced and highly promising strategy to overcome the anatomical and physiological barriers of the inner ear, particularly the round window membrane (RWM), for localized drug delivery. When integrated with poly(lactic-*co*-glycolic acid) (PLGA)—a widely used biodegradable and biocompatible polymer—this approach offers a dual advantage: magnetically guided navigation and controlled, sustained drug release.^61–63^

PLGA nanoparticles can be engineered to encapsulate both therapeutic agents and superparamagnetic iron oxide nanoparticles (SPIONs) or similar magnetically responsive materials. These hybrid magnetic PLGA nanocarriers can be administered into the middle ear cavity, typically near the RWM. Upon application of an external magnetic field over the temporal bone, the magnetic force directs and retains the nanoparticles at the cochlear interface, significantly improving their transmembrane penetration and accumulation in the perilymph.^64,65^

This magnetically driven targeting addresses one of the main challenges of inner ear therapy—low permeability across the RWM and rapid clearance from the middle ear. By enhancing local retention and ensuring sustained drug availability through the PLGA matrix, this technique minimizes systemic exposure, reduces dosing frequency, and improves therapeutic efficacy. Moreover, PLGA's tunable degradation profile allows for precise control over the drug release kinetics, ranging from days to weeks, depending on the formulation parameters (*e.g.*, lactic-to-glycolic acid ratio, molecular weight).^66,67^

Recent preclinical studies have demonstrated that magnetic PLGA nanoparticles can successfully deliver a range of therapeutic compounds—including corticosteroids, neurotrophic factors, and antioxidants—into the cochlea, with enhanced auditory protection and functional recovery, as measured by auditory brainstem response (ABR) and histological evaluation. Additionally, such systems can be further modified with surface ligands to improve RWM adhesion, reduce off-target interactions, or promote cell-specific uptake within cochlear structures.^68,69^ Despite its promise, magnetic PLGA nanoparticle-based delivery for cochlear applications is still in experimental stages.^70^ Challenges remain in optimizing particle size, magnetic content, targeting efficiency, and magnetic field strength for safe human application. However, this platform holds considerable translational potential for minimally invasive, site-specific treatment of sensorineural hearing loss, ototoxicity, and other cochlear pathologies, ushering in a new era of nanomedicine for auditory healthcare.^71,72^

For example, Du *et al.* (2013) demonstrated a pioneering application of magnetic targeting for cochlear drug delivery. In their study, PLGA-dex-Ac nanoparticles containing dexamethasone and iron oxide were intratympanically administered to guinea pigs.^73^ When an external magnet was applied adjacent to the RWM for one-hour post-injection, there was a marked increase in dexamethasone concentration in the perilymph, which remained elevated for several days compared to control groups. This sustained presence of the therapeutic compound strongly indicated that magnetic guidance significantly enhanced nanoparticle adhesion and penetration across the RWM, ensuring a prolonged therapeutic window within the cochlea. Importantly, Du and colleagues confirmed that the magnetic field did not compromise cochlear histology, thus affirming the safety and feasibility of this approach.

The translational implication of this study is profound. Magnetic nanoparticles embedded within PLGA can be actively directed across the RWM, bypassing the limitations of passive diffusion and mucosal clearance. Furthermore, the ability to modulate the duration and orientation of magnetic exposure provides a non-invasive method to control distribution gradients within the cochlea, which is critical in achieving localized drug concentrations near the sensory hair cells or spiral ganglion neurons.

Dormer *et al.* (2008) further validated this concept using technetium-99 m-labeled magnetic PLGA nanospheres.^74^ Following intratympanic administration and magnetic field application in guinea pigs, they observed over a threefold increase in radioactivity in the perilymph compared to non-magnetic controls. This quantifiable enhancement in cochlear uptake emphasized the effectiveness of magnetic fields in concentrating nanoparticles at the RWM and promoting their translocation into the inner ear. Dormer's work not only supported earlier findings but introduced a novel quantitative imaging framework for monitoring NP migration and bio-distribution in real time—a key feature in clinical translation.

Shirvalilou *et al.* (2018)^75^ and Cui *et al.* (2016)^76^ extended the application of magnetic PLGA nanoparticles into glioma-targeted delivery, employing superparamagnetic iron oxide nanoparticles (SPIONs) within PLGA cores and external magnetic fields to enhance tumor-specific delivery. Shirvalilou's study integrated PLGA with nano-graphene oxide and loaded the system with iododeoxyuridine (IUdR), a radiosensitizer. The magnetically guided platform significantly increased therapeutic localization and survival in rats. Similarly, Cui and colleagues utilized T7 peptide-functionalized magnetic PLGA nanoparticles for co-delivery of paclitaxel and curcumin, achieving 10-fold higher cellular uptake and improved blood–brain barrier penetration.

While these studies focused on the brain, their methodologies provide direct insight into improving cochlear delivery. Specifically, their success in overcoming barriers such as the blood–brain barrier (BBB)—which shares functional parallels with the RWM—suggests that similar ligand–magnet combinations could be tailored for cochlear delivery. For instance, peptides targeting cochlear-specific markers (*e.g.*, prestin or myosin VIIa) could be conjugated to magnetic PLGA carriers, while a magnet applied to the mastoid region would facilitate site-specific nanoparticle accumulation.

Wen *et al.* (2014) reinforced this approach by developing magnetic PLGA/lipid NPs decorated with the TAT peptide.^77^ The TAT-modified carriers exhibited enhanced uptake in brain endothelial cells under magnetic influence, confirming the synergy between magnetic guidance and cell-penetrating ligands. In otologic contexts, substituting TAT with cochlea-specific ligands could provide dual functionality—magnetically-assisted transport across the RWM and receptor-mediated internalization into target cells, such as outer hair cells or supporting cells.

Similarly, Mosafer *et al.* (2017) demonstrated the utility of aptamer-functionalized magnetic PLGA nanocarriers for doxorubicin delivery.^78^ The system, modified with AS1411 aptamer, achieved enhanced cellular specificity and allowed for simultaneous imaging and therapy. The potential for such platforms in cochlear applications lies in their theranostic capacity: imaging-guided therapy using aptamers that target cochlear proteins could revolutionize inner ear diagnostics and treatment.

#### 3.2.2 Ultrasound-enhanced permeability: a synergistic ally

Although less explored in otology, low-intensity focused ultrasound (LIFU) presents a powerful modality to temporarily increase membrane permeability without causing structural damage. In other tissue systems, ultrasound has been shown to open tight junctions, enhance endocytosis, and even facilitate transmembrane delivery of nanoparticles. Extrapolating this to the RWM, ultrasound could be used to transiently increase permeability, allowing PLGA nanoparticles to pass through more readily and in higher quantities.^79,80^

When combined with magnetic targeting, this presents a compelling two-tiered strategy: ultrasound to enable entry and magnetism to control destination. For instance, ultrasound can create transient openings in the RWM, during which magnetic PLGA NPs can be directed through these gaps using an external magnetic field. Once inside the perilymph, these NPs can be retained at desired cochlear locations by sustained magnetic attraction, ensuring site-specific release of encapsulated therapeutics.^80,81^

Moreover, ultrasound may enhance not just RWM penetration but also intracochlear dispersion and tissue uptake. Nanoparticles tend to aggregate or settle without external stimuli, but ultrasound waves can maintain them in suspension and improve distribution along the cochlear spiral. This would be particularly valuable for uniform drug distribution in cases such as bilateral sensorineural hearing loss or diffuse cochlear inflammation.^81,82^

#### 3.2.3 Integrative outlook: toward precision cochlear nanotherapy

Together, the above studies construct a powerful narrative: PLGA nanoparticles, when combined with magnetic or ultrasound-enhancing strategies, transcend the limitations of passive intratympanic delivery. The convergence of magnetic guidance and ultrasound modulation introduces a paradigm shift—from passive, diffusion-limited models to active, externally controllable, site-specific platforms. Such strategies allow for higher perilymphatic concentrations, sustained release kinetics, and reduced systemic toxicity, all of which are essential for treating delicate cochlear pathologies such as cisplatin-induced ototoxicity, aminoglycoside damage, age-related hearing loss, or autoimmune inner ear disease.

While Du *et al.* (2013)^73^ and Dormer *et al.* (2008)^74^ demonstrated the feasibility of magnetic cochlear targeting *in vivo*, and Cui *et al.* (2016),^76^ Wen *et al.* (2014),^77^ and Shirvalilou *et al.* (2018)^75^ illustrated its potential in overcoming biological barriers in oncology, the pathway toward otologic translation is now clearly visible. The next step involves the rational design of cochlea-specific, magnetically responsive PLGA nanoparticles, possibly combined with ultrasound-responsive materials, to realize a new era of nanomedicine for inner ear disorders. Integrating magnetic field navigation and ultrasound-facilitated permeation into PLGA-based nanoparticle design opens transformative possibilities for precision medicine in otology. The evidence across these studies justifies a concerted push toward multi-modal, controllable nanocarrier systems, offering new hope for diseases previously deemed inaccessible by systemic therapies. As technology advances, these synergistic delivery strategies promise to unlock the full therapeutic potential of PLGA nanocarriers in protecting and regenerating the intricate structures of the cochlea.

### 3.3 Systemic *vs.* local administration

Systemic *versus* local administration strategies for inner ear drug delivery represent two fundamentally distinct paradigms that have been extensively explored, particularly in the context of overcoming the biological and anatomical hurdles presented by the blood-labyrinth barrier (BLB). The BLB, much like the blood–brain barrier, presents a formidable obstacle for systemically administered therapeutics, severely restricting the passage of drugs into the cochlear perilymph and thereby limiting the efficacy of treatments aimed at sensorineural hearing loss. Multiple studies, including those by Hao and Li (2021),^70^ Xiao *et al.* (2021),^45^ and Moatti *et al.* (2023),^46^ have highlighted the limitations of systemic administration routes. Systemically delivered drugs often achieve subtherapeutic concentrations within the inner ear, and there is a concomitant risk of systemic toxicity or off-target effects. For instance, Xiao *et al.* (2021) emphasized how the isolated anatomical position of the inner ear, coupled with the limited vascularization from the labyrinthine artery and the restrictive nature of the BLB, makes systemic routes largely inefficient for cochlear targeting.^45^

In contrast, local administration, particularly *via* the intratympanic (IT) route, offers a promising and clinically translatable alternative. This strategy leverages direct access to the middle ear and allows therapeutics—especially nanoparticle-based systems—to permeate the round window membrane (RWM) and reach the cochlea. Hao and Li (2021) succinctly describe the IT route as a minimally invasive approach that allows drugs to diffuse across the RWM into the scala tympani, bypassing the BLB entirely.^70^ Moatti *et al.* (2023) provided further experimental backing using an ex vivo porcine RWM model that mimics the human membrane.^46^ Their work validated the IT route as a viable pathway for local drug administration while demonstrating the limitations of passive diffusion across the RWM, thereby motivating the need for enhanced delivery strategies.

This is where nanoparticle-based drug delivery systems—particularly poly(lactic-*co*-glycolic acid) (PLGA) nanoparticles—have garnered substantial attention. PLGA NPs are renowned for their biocompatibility, biodegradability, and controlled drug release profiles. However, the key challenge in local delivery remains the short residence time of the therapeutics in the middle ear cavity, which can lead to suboptimal drug permeation through the RWM. To address this, researchers have developed hydrogel-based reservoirs and thermosensitive polymeric systems designed to enhance retention at the RWM. These formulations enable sustained release of PLGA NPs at the site of administration, increasing the likelihood of successful RWM permeation and subsequent cochlear delivery.

The comparative advantage of these localized retention systems is evident when juxtaposed against systemic routes. For example, Xiao *et al.* (2021) emphasized the need for localized drug carriers that not only prolong residence time but also enhance targeted delivery through specific interactions with cochlear tissues.^45^ Hydrogel and thermoresponsive systems—often composed of polymers like poloxamers, gelatin, or chitosan—form viscous gels at body temperature, anchoring the PLGA NPs at the RWM interface. This strategy minimizes clearance *via* the Eustachian tube and allows a slow and sustained diffusion of drug-loaded NPs through the RWM. The benefits of this approach are underscored in studies involving cochlear protection against cisplatin or aminoglycoside-induced ototoxicity, where timely and sustained delivery of antioxidants (like edaravone or resveratrol) or anti-apoptotic agents (such as dexamethasone) through locally administered PLGA carriers proved far more efficacious than systemic alternatives.

Moreover, while systemic administration is largely passive and non-selective, local strategies are increasingly being refined with targeted modalities. Magnetic targeting and ultrasound enhancement, for instance, are two evolving technologies that provide spatial and temporal control over NP localization and penetration. These modalities complement the localized delivery by actively guiding PLGA NPs toward and across the RWM, overcoming one of the principal limitations of passive retention-based strategies.

In comparative terms, while systemic delivery might still hold relevance for diseases affecting both the ear and other organs or for patients unable to undergo IT procedures, its inefficiency for cochlear-specific treatment makes it a suboptimal choice for most inner ear conditions. Local delivery strategies—especially those combining prolonged retention, penetration enhancement, and active targeting—are now clearly emerging as the superior modality, particularly when using PLGA-based nanoparticle systems. The cumulative body of work from Hao and Li (2021),^70^ Xiao *et al.* (2021),^45^ and Moatti *et al.* (2023),^46^ along with emerging technological innovations, continues to support this paradigm shift, paving the way for safer, more effective, and precisely controlled inner ear therapies.

## 4. PLGA nanoparticles for otoprotection

### 4.1 Protection against ototoxic drugs

Cisplatin and aminoglycosides, including gentamicin and kanamycin sulfate (KS), are cornerstone chemotherapeutics and antibiotics with well-documented efficacy but notorious ototoxic side effects. These drugs exert their cytotoxicity largely through oxidative stress, triggering the overproduction of reactive oxygen species (ROS), mitochondrial dysfunction, and subsequent apoptosis in the inner ear's hair cells. In an effort to mitigate such damage, an expanding body of research has explored the use of poly(lactic-*co*-glycolic acid) (PLGA) nanoparticles (NPs) as a drug delivery vehicle to carry otoprotective agents directly to the inner ear. These systems offer the benefits of sustained release, reduced systemic toxicity, and targeted delivery, thereby enhancing therapeutic efficacy and safety profiles.

Mustafa *et al.* (2017) addressed the hydrophilic nature and short half-life of KS by encapsulating it in PLGA-vitamin E-TPGS NPs and further modifying them with polyethylene glycol (PEG) and water-soluble chitosan (WSC).^83^ This surface-engineered formulation—KS-PEG-WSC NPs—achieved significantly prolonged blood circulation and decreased nephrotoxicity and ototoxicity, demonstrating how nanoparticle architecture can directly influence systemic distribution and organ-specific toxicity. In the same vein, Park *et al.* (2022) leveraged superparamagnetic iron oxide nanoparticles (SPIONs) and dexamethasone (Dexa) co-loaded into PLGA NPs to guide drug delivery to the cochlea under a magnetic field.^84^ This magnetic targeting not only enhanced localization but dramatically improved auditory outcomes in ototoxicity-induced models, highlighting a breakthrough in minimizing systemic exposure while maximizing inner ear protection.

Complementing these approaches are anti-apoptotic and antioxidant strategies embedded in PLGA systems. Xiao *et al.* (2023) developed dual-responsive composite nanoparticles (CCC@mPP NPs) co-loaded with panax notoginseng saponins, tanshinone IIA, and ammonia borane, capable of responding to acidic pH and elevated ROS in the inner ear microenvironment.^85^ These NPs demonstrated near-complete preservation of hair cells in cisplatin- and aminoglycoside-induced hearing loss models, providing a compelling case for multifactorial protection *via* combinatory therapeutics. Similarly, Chen *et al.* (2022) designed curcumin-loaded PLGA NPs with chitosan coating (CUR-CS/PLGA NPs), showing superior cochlear accumulation and otoprotection compared to free curcumin or uncoated NPs, attributable to both sustained release and improved cellular uptake.^95^ These effects were corroborated by inhibition of pro-apoptotic proteins like Caspase-3 and Bax, and suppression of mitochondrial dysfunction in hair cells.

The promise of PLGA-based systems extends beyond traditional antioxidants and corticosteroids. Hong *et al.* (2023) utilized a Schwann cell model to assess the cytotoxicity of cisplatin and gentamicin and demonstrated that N-acetylcysteine (NAC)-loaded PLGA microparticles could moderately enhance Schwann cell viability under cisplatin stress, even though protective consistency varied.^86^ This highlights the potential for PLGA vehicles to explore novel therapeutic targets like Schwann cells, which are also implicated in ototoxic injury.^87^ Furthermore, Poudel *et al.* (2024) showed that a single subconjunctival injection of PLGA-DSP NPs (dexamethasone sodium phosphate) significantly mitigated corneal inflammation and neovascularization caused by nitrogen mustard—a model paralleling the inflammatory milieu seen in inner ear damage.^88^ This study reinforces the broader utility of sustained corticosteroid delivery *via* PLGA NPs for long-term protection against chemically induced injuries.

Cai *et al.* (2017)^42^ and Guo *et al.* (2022)^89^ provided additional evidence for the biocompatibility and inner ear permeability of PLGA systems. Cai's work confirmed that nanoparticle size, surface chemistry, and incorporation of cell-penetrating peptides (*e.g.*, low molecular weight protamine) critically enhance cochlear entry and retention, without inducing pathological changes.^42^ Guo's *in vivo* studies affirmed the safety of chitosan-modified PLGA NPs (CS-PLGA-NR NPs) in rat cochlear tissues, showing no inflammatory response or structural disruption post-tympanic administration.^89^ Finally, Orekhova *et al.* (2022) illustrated how PLGA NPs enhance the bioavailability and antifungal efficacy of pterostilbene in resistant Aspergillus biofilms, drawing a broader conclusion about PLGA's potential to breach biological barriers—much like the round window membrane—to deliver active agents directly to pathological sites in the ear.^90^

Together, these studies form a compelling narrative: PLGA-based nanoparticles are emerging as versatile, tunable platforms capable of delivering a broad spectrum of protective agents—including antioxidants, anti-inflammatory drugs, siRNA, and antifungals—into the cochlea to mitigate drug-induced ototoxicity. Whether by enabling sustained drug release, enhancing tissue permeability, reducing systemic toxicity, or allowing combinatorial and stimuli-responsive release, PLGA NPs are redefining therapeutic strategies for sensorineural hearing loss.

### 4.2 Noise-induced hearing loss (NIHL)

Noise-induced hearing loss (NIHL) is a prevalent auditory disorder characterized by sensorineural damage initiated through oxidative stress and mitochondrial dysfunction. Central to the pathological cascade in NIHL is the overproduction of reactive oxygen species (ROS), leading to cellular damage in the cochlear hair cells, synapses, and auditory nerve fibers. Poly(lactic-*co*-glycolic acid) (PLGA) nanoparticles (NPs) have garnered significant attention in recent years as effective delivery systems for antioxidants, mitochondrial protectants, and genetic materials to combat these ROS-driven pathologies.^91,92^

The use of PLGA-based nanoformulations to deliver ROS scavengers such as curcumin or mitochondrial protectants has demonstrated a compelling therapeutic potential in preserving cochlear integrity. These strategies are rooted in the recognition that excessive ROS not only cause lipid peroxidation and protein damage but also impair mitochondrial DNA (mtDNA), which is essential for ATP generation and cellular homeostasis in cochlear cells.^93,94^ This mechanism was elaborately characterized in the work of Chen *et al.* (2022), who demonstrated that acoustic trauma leads to significant oxidative damage to mtDNA, thereby reducing mitochondrial gene expression and ATP levels in rat cochleae.^95^ They further highlighted the role of mitochondrial transcription factor A (TFAM), whose mtDNA-binding functionality is disrupted under oxidative stress.^95^ Importantly, systemic administration of the mitochondria-targeted antioxidant mito-TEMPO (MT) was shown to protect auditory function and cochlear structures by preserving TFAM-mtDNA interactions, restoring ATP production, and attenuating oxidative stress. This systemic antioxidant intervention points to a potential therapeutic convergence with local delivery strategies using PLGA NPs, where both aim to maintain mitochondrial integrity but *via* different routes of administration.

Complementing this mechanistic insight, Shih *et al.* (2021) investigated the inflammatory pathways co-activated by ROS during acoustic trauma.^96^ Their study focused on high-mobility group box 1 (HMGB1), a nuclear protein that acts as a potent proinflammatory mediator when released into the extracellular space under stress conditions. Through intraperitoneal administration of *anti*-HMGB1 antibodies prior to noise exposure, the authors were able to significantly reduce ROS formation, cochlear inflammation, and subsequent auditory threshold shifts. Although this study did not employ PLGA carriers, it reinforced the therapeutic value of targeting ROS and inflammation early during acoustic insult. In light of this, encapsulating HMGB1-neutralizing agents in PLGA NPs could provide an even more localized and sustained method of inhibiting inflammation-driven cochlear damage.

Going beyond mitigation, Du *et al.* (2018) took a regenerative approach by utilizing PLGA NPs to deliver small interfering RNA (siRNA) targeting the Hes1 gene in noise-injured guinea pigs.^97^ Hes1, a downstream effector of the Notch signaling pathway, inhibits hair cell regeneration. The siRNA-loaded PLGA NPs enabled controlled and sustained gene silencing, which led to remarkable recovery of hearing function and regeneration of outer hair cells (OHCs) across a wide cochlear range over nine weeks. This study not only underscores the therapeutic potential of PLGA NPs for gene therapy but also bridges a critical gap by coupling ROS mitigation with regenerative strategies. The results demonstrated that PLGA NPs are capable not only of protecting against hair cell loss but also of promoting new hair cell formation, thereby offering a multipronged solution to NIHL.

Historical yet foundational evidence for ROS involvement in NIHL was established in Yamasoba *et al.* (1999).^98^ They explored the use of the iron chelator deferoxamine mesylate (DFO) alone and in combination with the hydroxyl scavenger mannitol to attenuate ROS-induced cochlear injury in guinea pigs. Their findings indicated that chelation therapy significantly reduced auditory threshold shifts and outer hair cell loss. Interestingly, the inclusion of glial cell line-derived neurotrophic factor (GDNF) *via* osmotic pumps provided additive functional protection, though without synergistic morphological benefits. These findings point to a layered therapeutic opportunity wherein ROS scavenging, neurotrophic support, and anti-inflammatory strategies can be integrated for maximal cochlear preservation. The fact that these drugs were delivered either systemically or *via* osmotic pumps reflects an earlier era of delivery technology, one that can be significantly advanced with the tunable release and targeted delivery features of modern PLGA NPs.

When integrated, these studies collectively chart a comprehensive roadmap for treating NIHL using PLGA-based nanocarriers. They validate both the protective and regenerative potentials of targeting oxidative stress and mitochondrial dysfunction. While Chen *et al.*^95^ and Shih *et al.*^96^ emphasized oxidative stress as a primary driver of cochlear damage, Du *et al.*^97^ expanded the therapeutic scope by demonstrating how genetic modulation *via* PLGA NPs could reverse noise-induced cellular deficits. In contrast, Yamasoba *et al.*^98^ offered early proof-of-concept for ROS scavenging strategies, thereby laying the groundwork for nanoparticle-mediated delivery.

A key advantage of PLGA-based delivery is the ability to localize treatment at the round window membrane (RWM), thereby enhancing drug residence time while minimizing systemic side effects. This contrasts with the systemic administration approaches employed by Chen *et al.*^95^ and Shih *et al.*^96^ which, despite efficacy, may be limited by the blood-labyrinth barrier and off-target effects. By tailoring PLGA formulations to release antioxidants or siRNA in response to environmental triggers (*e.g.*, pH, temperature, ROS concentration), future therapies could achieve spatiotemporal control of drug release directly in the cochlea.

Taken together, these studies not only corroborate the central role of oxidative damage in NIHL but also highlight the multifaceted advantages of PLGA NP-mediated therapies. They demonstrate how PLGA NPs can serve as protective agents (*via* antioxidants), anti-inflammatory modulators (*via* antibody delivery), and even regenerative tools (*via* siRNA delivery), thereby positioning them as a cornerstone of future nanomedicine-based otological interventions.

## 5. PLGA nanoparticles in inner ear regeneration

### 5.1 Hair cell regeneration

Systemic routes for delivering therapeutics to the inner ear face formidable biological constraints, primarily due to the blood-labyrinth barrier (BLB)—a highly selective barrier that rigorously regulates the passage of substances from the systemic circulation into cochlear fluids such as the perilymph and endolymph.^99,100^ Functionally analogous to the blood–brain barrier, the BLB serves a critical role in preserving the ionic and metabolic stability of the cochlear environment, but it also severely restricts the entry of systemically administered pharmacological agents. As a result, only minimal concentrations of these agents actually reach target structures within the inner ear. This renders systemic delivery not only inefficient but also potentially hazardous due to increased risk of off-target accumulation and systemic toxicity, especially with agents requiring high plasma concentrations to achieve therapeutic levels in the cochlea.^100–102^

In contrast, local administration strategies have emerged as a superior approach, circumventing the limitations of the BLB and enabling the direct and sustained delivery of therapeutics to cochlear tissues. Among these, the application of biodegradable PLGA nanoparticles (NPs) embedded within thermosensitive hydrogels or gel reservoirs placed into the middle ear cavity has shown great promise. These delivery matrices, upon administration, transition from a liquid to a semi-solid state at body temperature, prolonging the residence time of encapsulated nanoparticles at the round window membrane (RWM). This anatomical structure—one of the few non-osseous openings into the inner ear—serves as a critical portal for passive nanoparticle diffusion into the scala tympani and perilymphatic spaces.^102^

Experimental evidence has validated the pharmacokinetic and pharmacodynamic advantages of such localized platforms. For instance, Kim *et al.* (2021) demonstrated that dexamethasone (DEX)-loaded PLGA nanoparticles incorporated within a poloxamer-based thermosensitive gel could remain localized in the middle ear cavity for up to 48 hours.^102^ This extended retention significantly enhanced cochlear uptake of the drug compared to aqueous solution formulations. Moreover, histological evaluations revealed no observable damage or inflammatory response within cochlear tissues, affirming the biocompatibility and safety of these locally administered nanocarrier systems.

The advantages of local PLGA-based nanoparticle delivery are further underscored by studies employing RNA interference-based strategies. In an innovative preclinical study, Du *et al.* (2018) developed PLGA nanoparticles encapsulating small interfering RNA (siRNA) targeting Hes1 (siHes1 NPs), a transcription factor implicated in suppressing hair cell regeneration.^97^ When locally applied to noise-damaged cochleae in adult guinea pigs, these siHes1-loaded nanoparticles facilitated hair cell regeneration and significant auditory recovery over a 9-weeks period, as measured by auditory brainstem response (ABR) testing. Importantly, these regenerative and functional benefits were achieved without systemic exposure, which would have been insufficient for such outcomes due to poor inner ear bioavailability.

Although direct cochlear application is most relevant in auditory research, similar principles have been validated in peripheral nerve regeneration studies.^103–105^ Also, systemic approaches are not entirely without merit. For instance, Chen *et al.* (2022) reported that systemic administration of the mitochondria-targeted antioxidant mito-TEMPO conferred partial protection against cochlear damage induced by acoustic overstimulation.^95^ However, systemic delivery of such agents remains limited by poor cochlear bioavailability and off-target exposure. Notably, mito-TEMPO's therapeutic impact—mediated by reductions in mitochondrial oxidative stress, mtDNA depletion, and TFAM loss—could potentially be enhanced by incorporating it into PLGA nanoparticles for localized cochlear delivery, thereby increasing its bioavailability at the target site while minimizing systemic burden.

In synthesis, a growing body of evidence points to the superior efficacy of locally administered PLGA-based drug delivery systems in overcoming the limitations posed by the BLB. Strategies involving thermosensitive gels, sustained-release reservoirs, and siRNA-loaded nanoparticles allow for precise, site-specific delivery, prolonged drug exposure, and minimization of systemic side effects. As such, these platforms significantly outperform traditional systemic routes and represent a transformative advance in the field of inner ear therapeutics.

### 5.2 Stem cell integration and guidance

Delivering stem cell-derived exosomes and microRNAs *via* PLGA-based scaffolds and nanoparticles offers a promising route to enhance integration and survival of transplanted cells within cochlear niches. Although cochlea-specific studies remain limited, foundational research provides strong support for extrapolating key principles.^106,107^ Surface-modified PLGA-PEI nanoparticles have been shown to stimulate increased exosome release from mesenchymal stem cells by enhancing cellular internalization and triggering autophagy-related pathways. Positively-charged PLGA-PEI nanoparticles (approx. 100 nm) were internalized *via* clathrin-mediated endocytosis, upregulating Rab7 and Beclin-1 and increasing exosomal markers CD63, CD9, CD81, while maintaining high MSC viability at moderate doses.^108–110^ This demonstrates the feasibility of using PLGA-based nanoparticles not only as carriers but also as modulators of exosome generation, an asset when wishing to harness stem cell-derived vesicles for therapeutic use.

Scaffold systems incorporating miRNA cargos offer another complementary strategy. Qi and colleagues fabricated bilayer nanofibrous scaffolds composed of an outer PLGA layer and an inner gelatin matrix that encapsulates miR-181a/b-1. Through electrospinning, these composite fibers enabled controlled release of miR-181a/b-1, which promotes differentiation in adipose-derived MSCs *via* the PTEN/PI3K/AKT pathway. This demonstrates that PLGA nanofibers can be engineered to provide biochemical guidance cues to transplanted stem cells, potentially enhancing survival and lineage commitment.^111–114^

Scaffolds that integrate exosomal delivery have also been explored extensively in non-cochlear settings. Research on PLGA scaffolds coated with polydopamine (pDA) to immobilize human adipose-stem-cell-derived exosomes showed significantly enhanced loading and more sustained release over eight days compared to simple adsorption, promoting bone regeneration in murine cranial defects.^115,116^ Similarly, exosomes encapsulated within PLGA-PEG-PLGA microspheres affixed to PLA scaffolds were shown to release exosomes in a sustained fashion (over 10 weeks) and support dental pulp stem cell-mediated repair. These delivery platforms highlight the ability of PLGA-based systems to preserve exosome integrity, control release kinetics, and deliver bioactive vesicles in a localized tissue environment.^117,118^

While these studies relate primarily to bone or peripheral nerve models, they collectively support the potential adaptation for cochlear stem cell therapies. PLGA-PEI nanoparticles could be tailored to enter cochlear cells or delivered intratympanically to deposit exosome-stimulative cues within the inner ear environment.^119,120^ Concurrently, miRNA-loaded PLGA nanofibers could provide both structural scaffolding and molecular differentiation signals to progenitor cells in damaged cochlear niches. Combining these approaches—stimulating exosome release from engineered stem cells and delivering miRNA-loaded PLGA scaffolds—could significantly enhance the integration and functional survival of transplanted cells in the cochlea.^121,122^

Although cochlea-specific data using PLGA nanomaterials remains sparse, the mechanistic evidence from MSC systems supports a translational strategy: use PLGA-PEI nanoparticles to boost exosome secretion, encapsulate or immobilize these exosomes within PLGA-based scaffolds, and deliver miRNA cues *via* PLGA nanofibrous matrices to guide cell differentiation.^121,122^ This multimodal platform holds promise for overcoming current challenges in cochlear regeneration by providing structural guidance, anti-apoptotic signalling, and controlled molecular cues essential to stem cell integration and survival.

## 6. Preclinical evidence and experimental successes

### 6.1 Animal models

Multiple preclinical studies using rodent models (mice, rats, and guinea pigs) have demonstrated the therapeutic efficacy of poly(lactic-*co*-glycolic acid) (PLGA) nanoparticles in managing inner ear disorders. PLGA nanocarriers encapsulating neuroprotective, anti-inflammatory, or regenerative agents have effectively preserved auditory function, as indicated by stable or improved auditory brainstem response (ABR) thresholds following ototoxic insult, acoustic trauma, or surgical procedures—highlighting their neuroprotective capacity.^17,97^

Histopathological examinations further revealed markedly reduced cochlear damage in treated animals, including decreased degeneration of sensory hair cells, reduced spiral ganglion neuron apoptosis, and diminished inflammation within cochlear structures. These outcomes are attributed to the sustained, localized drug release and the ability of PLGA nanoparticles to cross the round window membrane (RWM) and reach target cochlear regions.^17^

Additionally, PLGA-based therapies have been associated with the upregulation of regenerative and neurogenic biomarkers such as BDNF, Ki-67, Sox2, and Atoh1, suggesting enhanced proliferation and neuronal repair. Some models also utilized PLGA nanoparticles for siRNA or gene delivery, achieving successful modulation of genes critical for cochlear regeneration.^97^

**Fig. 9:**
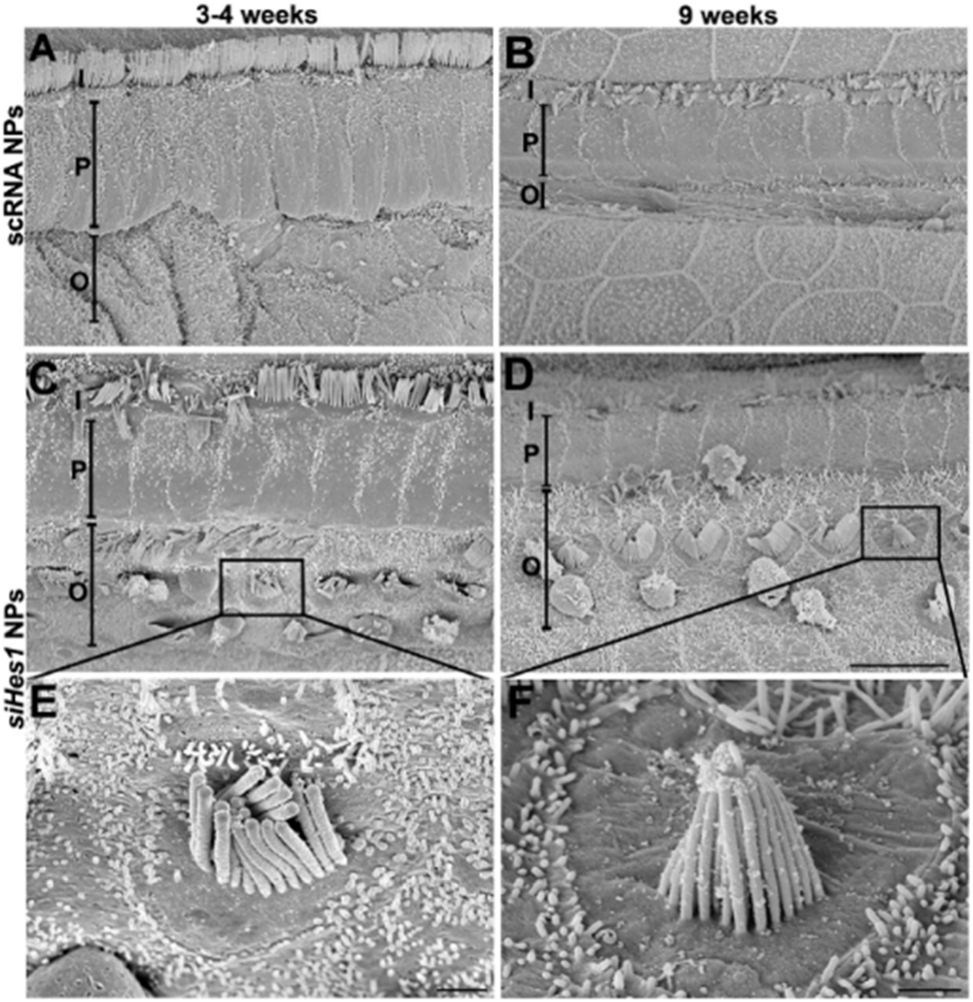
SEM images of OCs treated with siHes1 NPs show OHCs with immature stereocilia. (A and B) In scRNA NP-treated cochleae, OHCs were lost in the basal turns by 3 and 9 weeks. (C and D) siHes1 NP-treated ears retained many basal OHCs with proper positioning and mature stereocilia. Some OHCs displayed short, disorganized stereocilia lacking typical stair-step structure (C–F). I, P, and O indicate IHCs, pillar cells, and OHCs. Scale bars: 10 µm (D, for A–D); 1 µm (E and F).^97^ This figure is available under the Creative Commons CC-BY–NC–ND license and permits non-commercial use of the work as published, without adaptation or alteration provided the work is fully attributed.

A compelling example is presented by Du *et al.* (2018), who encapsulated siRNA targeting the Notch pathway effector *Hes1* into PLGA nanoparticles and administered them to guinea pigs after noise-induced hearing loss.^97^ Over nine weeks, animals treated with siHes1-loaded PLGA NPs showed significant restoration of ABR thresholds and regeneration of ectopic myosin VIIa-positive inner hair cells (IHCs) within the basal and second turns of the cochlea. Scanning electron microscopy confirmed the presence of immature outer hair cells (OHCs) with short, disorganized stereocilia (Fig. 9), and additional regenerating hair-cell-like structures exhibiting dense, irregular stereociliary bundles were identified in the basal turns of treated cochleae.

Mechanistically, acoustic trauma induced rapid *Hes1* upregulation within 24–48 hours, validating it as a therapeutic target. Treatment with siHes1-loaded PLGA NPs effectively suppressed *Hes1* expression, preserved supporting cell density—particularly in the 32 kHz region—and maintained epithelial integrity. These findings demonstrate that PLGA-based delivery of siHes1 promotes regeneration of both immature and mature cochlear hair cells, restores auditory function, and preserves cochlear cytoarchitecture. These preclinical studies establish PLGA nanoparticles as multifunctional platforms capable of conferring neuroprotection, enabling targeted gene modulation, and supporting structural regeneration of the inner ear.^97^

The study by Wang *et al.* (2023) provides compelling evidence for the efficacy of poly(lactic-*co*-glycolic acid) (PLGA) nanoparticles in protecting against ototoxic hearing loss, supporting the broader observation that PLGA NP-based therapies preserve auditory function, reduce histological damage, and enhance regenerative biomarkers in preclinical models.^17^ Addressing a major challenge in cochlear drug delivery—poor therapeutic distribution in the apical and middle turns due to anatomical and fluidic barriers—the authors functionalized PLGA NPs with A665, a peptide with high affinity for prestin, a protein selectively expressed in outer hair cells (OHCs). This modification enhanced both round window membrane (RWM) permeability and targeted cochlear uptake, particularly in regions that typically receive subtherapeutic exposure.

**Fig. 10:**
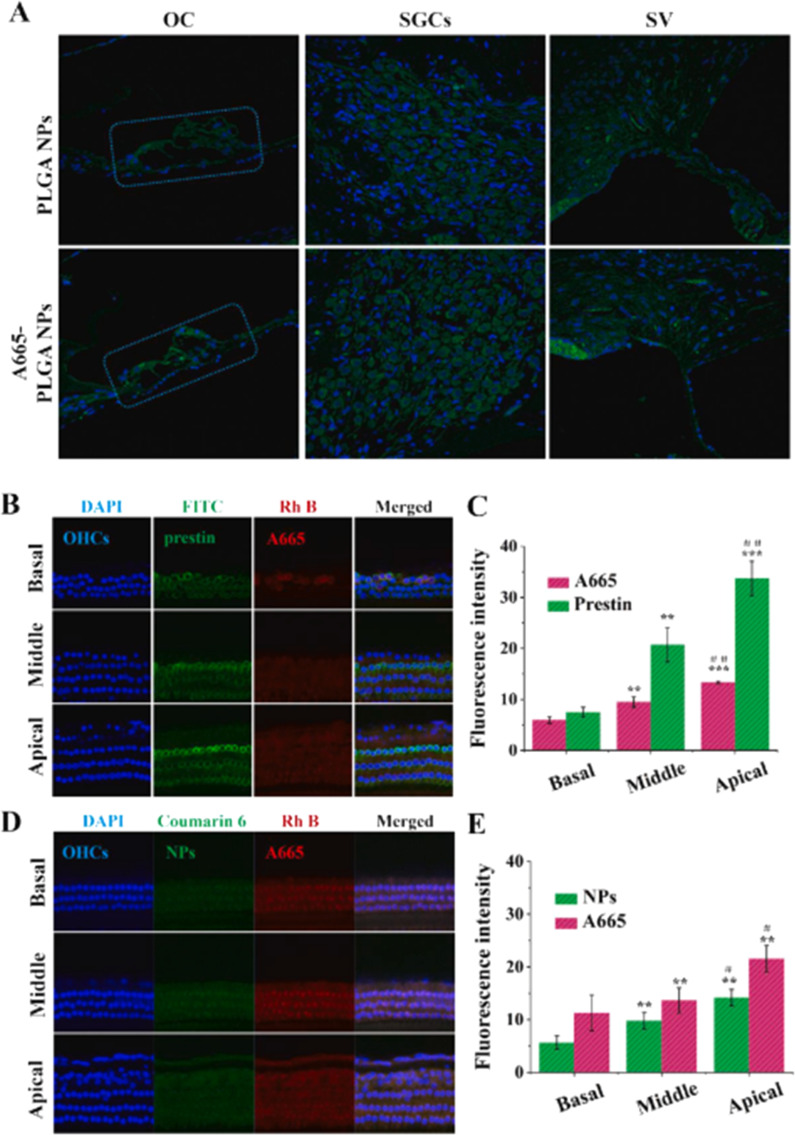
Targeted cochlear distribution of PLGA nanoparticles. (A) Localization of coumarin 6 (C-6)–loaded PLGA and A665-PLGA NPs in the stria vascularis (SV), organ of Corti (OC), and spiral ganglion cells (SGCs) after round window membrane (RWM) delivery (400×). (B) Colocalization of rhodamine B-labeled A665 with FITC-labeled prestin in outer hair cells across cochlear turns. (C) Quantitative analysis of colocalization in (B). (D) Distribution of C-6 and Rhodamine B-labeled A665-PLGA NPs in basal, middle, and apical turns. (E) Quantitative comparison of NP distribution in (D). Values are mean ± SD; **P < 0.01, ***P < 0.001 *vs.* basal; #*P* < 0.05, ##*P* < 0.01 *vs.* Middle.^17^ This figure is available under the Creative Commons CC-BY–NC–ND license and permits non-commercial use of the work as published, without adaptation or alteration provided the work is fully attributed.

As shown in Fig. 10, histological and fluorescence imaging revealed strong co-localization of A665 with prestin, indicating preferential accumulation of A665-PLGA NPs in OHCs across the basal, middle, and apical cochlear turns. Quantitative fluorescence data (Fig. 10A–E) confirmed improved NP navigation and distribution relative to non-functionalized PLGA NPs, overcoming the diffusion limitations of perilymphatic transport. Importantly, the A665 conjugation remained stable during cochlear transit, ensuring functional peptide integrity—a key factor for translational viability.

Therapeutic performance was validated in a kanamycin-furosemide–induced guinea pig model of hearing loss, where curcumin (CUR) was encapsulated in A665-PLGA NPs. As demonstrated, CUR/A665-PLGA NPs achieved near-complete OHC protection and superior auditory recovery compared with free CUR or non-targeted CUR/PLGA NPs. OHC survival reached approximately 99% in the apical region, while ABR thresholds at 4 kHz, 8 kHz, and click stimuli were restored to near-normal levels, indicating robust functional recovery rarely achieved with conventional local therapy.

Biocompatibility analyses confirmed the system's safety showing preserved cochlear architecture—including the stria vascularis, spiral ganglion, and organ of Corti—without signs of inflammation or cellular loss. ABR responses remained within normal thresholds, and zebrafish embryonic assays revealed no developmental toxicity.

Wang *et al.* (2023) demonstrate that prestin-targeted A665-PLGA nanoparticles effectively surmount cochlear delivery barriers, enhance OHC-specific uptake, and prevent drug-induced auditory damage. These findings substantiate the promise of rationally engineered PLGA nanocarriers as a safe and potent platform for localized cochlear therapy and long-term otoprotection.^17^

In the study by Zhao *et al.* (2023), curcumin and cisplatin were co-delivered *via* borneol/R8dGR peptide-modified PLGA NPs to overcome the blood–brain barrier (BBB) and target pediatric brainstem glioma (PBSG).^123^ Intranasal delivery of these NPs not only improved brain accumulation but also mitigated tumor hypoxia and angiogenesis—key contributors to tissue degeneration and impaired neurological signaling.^124^ While ABR was not directly measured, the extended survival of tumor-bearing mice and reduced pathological burden strongly indicate preserved neural integrity, akin to functional protection that would reflect in preserved ABR. Furthermore, the modulation of tumor microenvironment suggests an indirect enhancement of regenerative potential through reduced stress and hypoxia-related signalling.

Alonso *et al.* (2025) demonstrated the successful targeting of hippocampal and cortical brain regions with glutathione- and phenylalanine-functionalized PLGA NPs in rats.^125^ These nanoparticles bypassed the BBB and maintained localization in critical neuroanatomical areas for at least two hours, resisting *P*-glycoprotein-mediated efflux. Importantly, no histological damage was observed, suggesting the biocompatibility and non-toxicity of the NPs. Although this study did not involve a disease model or ABR measurement, the preserved tissue architecture, BBB penetration, and prolonged brain residence lay the groundwork for enhanced neuroregenerative potential through improved pharmacokinetics and targeted delivery.

Xu *et al.* (2017) provided a direct assessment of regeneration in a peripheral nerve model using PLGA membranes coated with NECL1 to promote Schwann cell adhesion and growth.^125^*In vivo*, these conduits significantly enhanced Schwann cell proliferation, neurotrophic factor expression (GDNF, BDNF, CNTF), and functional recovery of transected sciatic nerves. These regenerative markers are equivalent to molecular indicators of neural repair and parallel those used to infer auditory protection in ABR-related studies. The restoration of Schwann cell activity and nerve continuity strongly supports the potential of PLGA-based systems in preserving function following nerve damage.

In the case of Godse *et al.* (2025), co-encapsulation of the antiretroviral drug elvitegravir (EVG) and curcumin in PLGA NPs effectively targeted HIV CNS reservoirs.^126^ The delivery system reduced neuroinflammation and oxidative stress, while preserving neuronal markers such as NeuN, L1CAM, and synaptophysin. These markers are directly linked to functional neural integrity and by extension suggest preserved neural signaling akin to maintained ABR. The dual-action NPs not only modulated cytokine activity but also improved antioxidant defences, highlighting a synergy between functional protection and regenerative support within the CNS.

Kuo & Tsai (2018) expanded on neuroprotective outcomes by engineering PLGA-based nanocarriers functionalized with monoclonal antibodies and loaded with curcumin and rosmarinic acid.^127^ These targeted formulations reduced phosphorylation of tau protein and p38 MAPK in β-amyloid-insulted neuronal cultures—hallmarks of Alzheimer's disease progression. By reversing these pathological changes and preserving neuronal viability, the study implicates PLGA NPs in mitigating neurodegenerative histology and supporting functional capacity. While ABR was not assessed, the reduction in tau and p38 activation strongly aligns with mechanisms of auditory and central neuroprotection.

Saleh *et al.* (2024) directly addressed hippocampal histopathology and cognitive decline in a rat model of sporadic Alzheimer's disease using berberine-loaded PLGA-Tet1 NPs.^128^ These NPs restored markers of neuroplasticity (BDNF, pCREB), attenuated tau hyperphosphorylation, reduced pro-inflammatory cytokines, and improved cognitive performance. Histologically, hippocampal damage was markedly reduced, indicating preserved neural architecture. These findings demonstrate both histological and functional improvements that are translatable to auditory preservation paradigms like ABR, especially when considering the overlapping neurotrophic and anti-inflammatory pathways involved.

Madani *et al.* (2024) developed poloxamer-coated PLGA NPs loaded with methotrexate and paclitaxel to target glioblastoma.^129^ These NPs crossed the BBB efficiently, reduced tumor metabolic activity (as shown by PET), and lowered expression of Ki-67, a proliferation marker. Although not focused on ABR or hearing, the reduction in tumor burden, decreased neurotoxicity, and high brain localization of NPs mirror outcomes seen in auditory models where reduced pathological load correlates with preserved ABR. The histological protection and targeted drug delivery highlight a neuroprotective role for PLGA NPs.

Finally, Firouzi-Amandi *et al.* (2018) investigated PLGA-PEG NPs loaded with chrysin to modulate macrophage phenotype from pro-inflammatory M1 to anti-inflammatory M2.^130^ The shift in macrophage polarization was accompanied by reduced levels of TNF-α, IL-1β, and IL-6, while increasing M2 markers like Arg1 and Fizz. This immunomodulatory effect is relevant for auditory protection as excessive inflammation is a key driver of cochlear damage and ABR decline. By restoring immune balance, the study supports the regenerative and protective potential of PLGA NPs in neural environments. Across these diverse rodent studies, PLGA NPs consistently demonstrate efficacy in preserving neural function—either directly *via* ABR-equivalent outcomes or indirectly through reduced histological damage and enhanced regenerative signalling. Whether through modulating inflammation, enhancing BBB permeability, or promoting neurotrophic support, these systems exemplify a convergent therapeutic strategy that integrates functional protection with molecular regeneration.

### 6.2 Imaging and distribution studies

Fluorescently labelled PLGA nanoparticles (NPs) have been consistently demonstrated to localize within critical cochlear structures—the organ of Corti (OC), stria vascularis (SV), and spiral ganglion cells (SGCs)—following local administration, without triggering inflammation or toxicity. In early foundational research in guinea pigs, intratympanic deposition of rhodamine-loaded PLGA NPs on the round window membrane (RWM) resulted in rapid entry into scala tympani and sustained fluorescent signal within OC structures (including OHCs) over a two-hour timeframe, whereas systemic injection failed to achieve similar cochlear accumulation.^134,135^ In a more detailed comparative study, surface-modified PLGA NPs—coated with methoxy-PEG, poloxamer-407 (P407), or chitosan—were evaluated for cochlear penetration. Near-infrared imaging at 24 h post administration revealed that P407-PLGA NPs produced the highest cochlear fluorescence intensity across all three turns, notably in OC, SV, and SGCs, whereas chitosan-coated NPs showed minimal uptake; histological evaluations confirmed the absence of inflammatory or structural damage in these tissues.^42^ Importantly, the combination of P407-PLGA with a cell-penetrating peptide (LMWP) further enhanced cochlear uptake without inducing tissue pathology, reinforcing the biocompatibility and targeting efficiency of optimized PLGA NP formulations.^42,136^ Similarly, fluorescent PLGA NPs embedded within poloxamer-based thermosensitive gels achieved penetration into OC, SV, and SGCs in mice, with no signs of cellular toxicity or morphological alteration under histological analysis.^30^ Collectively, these imaging and biodistribution studies provide a robust body of evidence that fluorescently labelled PLGA nanoparticle systems, when administered *via* the middle ear, can effectively traverse anatomical barriers to reach and persist in cochlear sensory and neural compartments while maintaining excellent safety profiles.

### 6.3 Comparative studies with other nanocarriers

When benchmarked against alternative nanocarriers—such as liposomes or metallic nanoparticles—PLGA-based nanoparticles (NPs) consistently demonstrate superior safety, prolonged therapeutic duration, and enhanced inner ear bioavailability. In rodent cochlear studies, intratympanic administration of rhodamine-loaded PLGA NPs achieved measurable fluorescence within the scala tympani and organ of Corti (OC) within minutes, significantly outperforming systemic delivery in cochlear targeting.^131^ These PLGA NPs offered sustained drug exposure without evidence of sensory cell damage or immunological reaction. In comparison, liposome-based nanocarriers—despite their capacity for controlled release—have been shown to cause cytotoxicity when cationic lipid components are present; even neutral liposomes can subtly elevate inflammatory markers like hyaluronic acid, though animal ABR thresholds remain unaffected.^132^

Further head-to-head observations from comprehensive reviews indicate that SPION-based metallic nanoparticles may present risks: heavy metals can induce reactive oxygen species generation, oxidative stress, and mitochondrial disruption—factors absent in PLGA systems, which degrade safely *via* hydrolysis.^132,133^ In contrast, PLGA-PEG-PLGA thermosensitive gels loaded with corticosteroids or antiviral compounds enabled extended cochlear residence and controlled release—with no significant changes in ABR thresholds at recommended doses (*e.g.*, 0.05 mL), further confirming PLGA's excellent safety profile. Only higher volumes (*e.g.*, 0.1 mL) introduced mild threshold shifts, underscoring the importance of controlled dosing.^134^

Additionally, PLGA NPs embedded in thermosensitive chitosan hydrogels achieved prolonged drug retention in guinea pig cochleae, extending exposure several-fold compared to solution or hydrogel alone, without histological abnormalities or inflammatory infiltration across cochlear compartments.^137^ In the context of solid lipid nanoparticles (SLNs), *in vivo* assessments revealed that low-dose hydrocortisone encapsulated within SLNs protected auditory cells following cisplatin exposure without disturbing hearing thresholds or causing hair cell loss—yet SLNs often require surfactant formulations that risk dose-dependent cytotoxicity and have shorter release durations than PLGA carriers.^21^

Taken together, these comparative studies establish a clear hierarchy: PLGA nanoparticles, owing to their biodegradable and tunable design, deliver superior cochlear bioavailability, maintain prolonged therapeutic presence, and offer exceptional histocompatibility. In contrast, liposomal or metallic alternatives carry increased risks of inflammation, cytotoxicity, and inconsistent inner ear access, especially when high-dose or charged formulations are employed.

## 7. Challenges and limitations

Despite the promising progress in the development of PLGA-based nanoparticles (NPs) for targeted inner ear therapy, several critical challenges must be addressed before these systems can be widely adopted in clinical practice. These limitations span anatomical, physicochemical, immunological, and regulatory dimensions, each posing unique barriers to effective and scalable therapeutic deployment.^138,139^

### 7.1 Round window variability and permeability

One of the foremost anatomical challenges lies in the inter-individual variability of the round window membrane (RWM), which serves as a principal gateway for non-invasive nanoparticle delivery into the cochlea. The RWM exhibits significant differences in thickness, surface area, epithelial integrity, and composition among individuals, and even across species in preclinical models. These structural variations can markedly influence NP diffusion, permeability, and eventual bioavailability within target inner ear compartments. Moreover, pathological conditions—such as otitis media, fibrosis, or inflammation—can further compromise RWM permeability by altering the membrane's extracellular matrix or inducing fibrotic thickening. To overcome this bottleneck, researchers are investigating pre-treatment strategies involving safe permeation enhancers (*e.g.*, hyaluronidase or surfactants) that transiently disrupt tight junctions or increase membrane porosity. Concurrently, tailored particle designs—such as optimizing size, charge, and surface modifications (*e.g.*, PEGylation or ligand conjugation)—are being employed to maximize *trans*-RWM transport without compromising tissue integrity.^140–143^

### 7.2 Drug loading efficiency and stability

Another major limitation in PLGA-based delivery systems is the relatively low encapsulation efficiency and limited stability when formulating hydrophilic or highly water-soluble therapeutic agents. PLGA matrices naturally favor the encapsulation of lipophilic drugs due to their hydrophobic polymeric backbone, rendering the loading of small hydrophilic molecules, peptides, or nucleic acids inefficient and prone to burst release. This inefficiency compromises sustained release profiles and may necessitate higher dosing, increasing the risk of toxicity. To address this, advanced formulation techniques—such as the water-in-oil-in-water (W/O/W) double emulsion solvent evaporation method, nanoprecipitation, and electrostatic surface adsorption—are being explored to improve drug payloads. Some approaches also involve using polymer blends or incorporating hydrophilic additives (*e.g.*, PEG, PVA, or lecithin) to modulate matrix hydrophilicity and improve drug-polymer compatibility. Despite these efforts, batch-to-batch consistency in drug loading and release kinetics remains a significant formulation hurdle, especially under scaled-up production conditions.^144–146^

### 7.3 Immune responses and long-term safety

While PLGA is widely recognized for its biodegradability and biocompatibility—being approved by regulatory agencies such as the FDA and EMA—there is growing concern regarding its immunological safety, especially under chronic or repeated exposure scenarios. Upon degradation, PLGA breaks down into lactic and glycolic acids, which may induce local acidity and potentially lead to inflammation or cytotoxicity if not properly buffered. Moreover, repeated administration of NPs could provoke innate or adaptive immune responses, particularly if residual solvents, surfactants, or immunogenic surface moieties are present in the final formulation. Additionally, off-target accumulation of NPs in non-cochlear tissues, such as the cerebrospinal fluid or vestibular organs, raises concerns about systemic toxicity or unintended neuroinflammation. Therefore, rigorous long-term toxicological evaluations—including biodistribution profiling, histopathological analyses, and immune biomarker assessments—are essential prerequisites for clinical translation. Importantly, these studies must be conducted in multiple species and across varying dosing regimens to accurately capture potential interspecies immunological discrepancies.^147–150^

### 7.4 Regulatory and manufacturing hurdles

Translating PLGA-based cochlear nanotherapeutics from bench to bedside also faces formidable regulatory and manufacturing challenges. The complexity of nanoparticle synthesis—often involving multi-step processes such as emulsification, solvent evaporation, lyophilization, and surface functionalization—poses scalability issues. Achieving Good Manufacturing Practice (GMP)-compliant production with consistent physicochemical properties (*e.g.*, size, polydispersity, zeta potential, and drug loading) is technically demanding and resource-intensive. Furthermore, regulatory agencies require exhaustive characterization of raw materials, intermediates, and final products, as well as data on shelf-life stability, sterilization protocols, and packaging integrity. Additional challenges lie in harmonizing manufacturing standards across different regulatory jurisdictions and establishing validated assays for *in vitro* release, *in vivo* pharmacokinetics, and safety endpoints. Without robust and reproducible protocols, the path toward clinical trials and eventual market authorization remains obstructed.^151–155^

## 8. Future directions and translational outlook

As PLGA-based nanoparticles continue to show immense promise in preclinical studies for inner ear therapy, the horizon is now shifting toward translational applications that could redefine the management of hearing loss and other cochleopathies. Future research is expected to focus on optimizing clinical relevance, enhancing diagnostic integration, and advancing personalized and multimodal therapeutic strategies. Several key directions are poised to shape the next phase of innovation and clinical translation.

### 8.1 Clinical trials and human applications

To date, the clinical translation of nanoparticle-based therapeutics for inner ear disorders remains in its infancy, with only a limited number of early-phase human trials currently underway. These include evaluations of NP formulations for sustained steroid delivery or otoprotective agents in patients undergoing cochlear implantation or ototoxic drug therapy. A major bottleneck remains the anatomical and physiological gap between rodent models—commonly used in preclinical studies—and the human auditory system. Significant interspecies differences in cochlear size, RWM architecture, perilymph dynamics, and immune environment must be accounted for to ensure translational fidelity. Consequently, future research must emphasize the development of delivery systems specifically tailored to human anatomy, potentially involving 3D-printed cochlear models or ex vivo human temporal bone platforms. Moreover, patient-specific factors such as age, cochlear maturity, and disease phenotype necessitate customized therapeutic approaches—particularly for pediatric *versus* adult populations. Pediatric patients, for example, may benefit from lower dosages, alternative delivery vehicles, or age-appropriate device integration to accommodate developmental anatomy and pharmacokinetics.

### 8.2 Theranostics and personalized medicine

The integration of therapeutic and diagnostic functionalities—commonly referred to as *theranostics*—represents a powerful future direction in inner ear nanomedicine. PLGA nanoparticles offer a versatile platform to co-deliver drugs alongside imaging agents such as gadolinium-based MRI contrast agents, near-infrared (NIR) dyes, or radionuclides. This dual capability allows for real-time monitoring of drug delivery, biodistribution, and treatment efficacy, significantly enhancing clinical decision-making and safety. Furthermore, PLGA matrices can be engineered to respond to physiological stimuli within the cochlea, such as pH shifts, enzyme activity, or oxidative stress. These “smart” or stimuli-responsive systems could enable on-demand, site-specific drug release, minimize off-target effects and improving therapeutic precision. In the context of personalized medicine, this opens the possibility of tailoring release profiles, dosing regimens, and therapeutic payloads based on patient-specific cochlear biomarkers or genetic profiles, ensuring more effective and individualized interventions.

### 8.3 Integration with gene editing and CRISPR technologies

One of the most transformative frontiers in cochlear therapeutics lies in the potential application of gene-editing technologies—particularly CRISPR-Cas9—for the correction of inherited genetic mutations underlying congenital or progressive hearing loss. However, the successful clinical implementation of CRISPR hinges on the development of safe, efficient, and tissue-specific delivery vehicles. PLGA nanoparticles have emerged as attractive non-viral vectors for delivering gene-editing components, including Cas9 ribonucleoprotein complexes, guide RNAs, and donor DNA templates, directly to cochlear hair cells or supporting cells. Their biodegradable nature, tunable surface properties, and reduced immunogenicity offer advantages over traditional viral vectors, particularly for transient gene modulation or multiplexed editing. Nonetheless, this application is still in its nascent stage, and substantial challenges remain—including achieving endosomal escape, nuclear localization, and cell-type-specific uptake within the cochlea. Ongoing research is expected to focus on refining nanoparticle formulations for efficient CRISPR delivery, minimizing off-target effects, and validating gene-editing outcomes through both molecular and auditory functional assessments.

### 8.4 Multimodal therapy platforms

The future of inner ear therapy is likely to move beyond monotherapy toward integrative, multimodal treatment paradigms that leverage the synergistic benefits of various interventions. PLGA-based nanoparticles can be combined with cochlear implants to deliver anti-inflammatory or neurotrophic factors that improve electrode-neuron interface and preserve residual hearing. Similarly, the co-delivery of PLGA NPs with stem cells or exosomes may enhance cellular survival, differentiation, and tissue regeneration in sensorineural hearing loss. Additionally, incorporating electrophysiological stimulation or bioelectronic interfaces alongside nanoparticle-based drug release systems could potentiate synaptic repair and neuroplasticity. These hybrid approaches offer particularly exciting prospects for severe or congenital forms of hearing loss, where monotherapies often fall short. However, the success of such platforms will depend on precise coordination of drug kinetics, device integration, and biocompatibility across multiple therapeutic modalities. Interdisciplinary collaborations between nanotechnologists, audiologists, neurosurgeons, and bioengineers will be essential to bring these complex but highly promising systems into clinical reality.

## 9 Conclusion

Poly(lactic-*co*-glycolic acid) (PLGA)-based nanoparticles have emerged as one of the most promising platforms for overcoming the major barriers to inner ear drug delivery. Their tunable physicochemical properties—particularly controllable size, zeta potential, and surface functionalization—enable targeted and sustained therapeutic release within the cochlea. These attributes allow for efficient *trans*-round window membrane transport, enhanced cellular uptake, and minimized systemic exposure. Recent innovations, including magnetic field–assisted and ultrasound-enhanced delivery, have further improved the localization and retention of PLGA nanocarriers in cochlear tissues. Preclinical studies consistently demonstrate their potential to prevent ototoxic and noise-induced damage while promoting hair cell regeneration and neural repair. Despite this progress, challenges such as variable round window permeability, limited drug loading capacity, potential immunogenicity, and translational regulatory constraints remain. Addressing these limitations through optimized formulation design, advanced modeling, and standardized evaluation protocols will be critical to advancing clinical adoption. Looking forward, the integration of PLGA nanoparticle systems with gene-editing technologies, theranostic tools, and personalized medicine approaches offers a clear path toward next-generation therapies. PLGA-based nanomedicine represents a cornerstone in the evolving landscape of otoprotection and inner ear regeneration, with the potential to transform current treatment paradigms for sensorineural hearing loss.

## Ethics approval

This study did not involve human participants or animals and therefore did not require ethical approval from an institutional or national ethics committee.

## Human and animal rights declaration

This article does not contain any studies with human participants or animals performed by any of the authors. All relevant institutional, national, and international guidelines were adhered to, where applicable.

## Author contributions

Olusegun Oluwaseun Jimoh conceptualized the study and led the overall coordination and supervision of the review. Tolulope Ajuwon, Somtochukwu Samuel Okonkwo, and Raymond Femi Awoyemi contributed to literature search, data curation, and initial manuscript drafting. Ibukunoluwa Olaosebikan, Olatayo Adedayo Olahanmi, and Christopher Mbonu contributed to critical analysis, synthesis of reviewed literature, and writing—original draft preparation. Idris Oladimeji Junaid participated in writing—review and editing and contributed to visualizations and formatting. Ikenna Odezuligbo and Kristinoba Olotu provided intellectual input, validation of sources, and manuscript refinement. Ikhazuagbe Hilary Ifijen conceptualized the study and served as the senior author and supervisor, providing guidance, critical review, and final approval of the manuscript. All authors have read and approved the final version of the manuscript.

## Conflicts of interest

The authors declare that they have no competing financial or non-financial interests.

## Data Availability

No new data were generated or analyzed in this study. All data supporting the findings of this review are available within the manuscript and the references cited.
